# Dietary Paper Mulberry Silage Supplementation Improves the Growth Performance, Carcass Characteristics, and Meat Quality of Yangzhou Goose

**DOI:** 10.3390/ani14030359

**Published:** 2024-01-23

**Authors:** Ruhui Wang, Xin Wang, Yi Xiong, Jingwen Cao, Luiz Gustavo Nussio, Kuikui Ni, Yanli Lin, Xuekai Wang, Fuyu Yang

**Affiliations:** 1 College of Grassland Science and Technology, China Agricultural University, Beijing 100193, China; wangruhui9288@163.com (R.W.); wangxin1814@126.com (X.W.); caojingwen1207@163.com (J.C.);; 2 College of Animal Science, Guizhou University, Guiyang 550025, China; 3 Department of Animal Sciences, Luiz de Queiroz College of Agriculture, University of Sao Paulo, Piracicaba 13418-900, Brazil; nussio@usp.br

**Keywords:** goose, paper mulberry, growth performance, carcass characteristic, meat quality

## Abstract

**Simple Summary:**

Paper mulberry (PM) is an unconventional protein feed material, and silage is its main processing method. The present study aimed to investigate the health benefits and meat quality of supplementing Yangzhou geese with paper mulberry silage. Results indicated that paper mulberry silage supplementation had a promotional effect on the growth of Yangzhou geese, the sensory quality of the breast muscles improved, and the nutritional quality enhanced. Untargeted metabolomics analysis demonstrated that PM treatment enhanced guanidinoacetic acid levels in breast muscles and facilitated the metabolism of amino acids and the role of substances in lipid antioxidant pathways. Consequently, paper mulberry could be considered to be a novel protein feed option capable for geese.

**Abstract:**

There have been few investigations into the health benefits and meat quality of supplementing Yangzhou geese with paper mulberry silage. One hundred and twenty 28-day-old Yangzhou geese were selected for the experiment and randomly divided into two groups: a control group (CON) and a paper mulberry silage group (PM), with six replicates in each group. The experiment lasted for a total of 6 weeks. The experiment found that compared with CON, PM had a promoting effect on the average daily weight gain of Yangzhou geese (*p* = 0.056). Sensory and nutritional analysis of breast muscles revealed a decrease in a* value (*p* < 0.05) and an increase in protein content (*p* < 0.05) following PM treatment. Through untargeted metabolomics analysis of breast muscle samples, it was found that 11 different metabolites, including guanidinoacetic acid and other substances, had a positive effect on amino acid metabolism and lipid antioxidant pathways of PM treatment. Overall, the strategy of feeding Yangzhou geese with paper mulberry silage is feasible, which can improve the sensory quality and nutritional value of goose meat. The experiment provides basic data for the application form of goose breeding, so exploring the impact of substances within paper mulberry on goose meat should be focused on in the future.

## 1. Introduction

Goose (*Anas cygnoides*) meat has been recognized for its potential health benefits due to its high-quality protein and unsaturated fatty acid [1,2]. In 2020–2021, there was around 700 million producing animals/slaughtered (globally around 750 million) and the volume of goose meat (fresh or chilled) exceeded 4 million tons in China according to FAOSTAT statistical data. Geese, being herbivorous poultry, have a natural inclination towards consuming grass and possess the ability to digest various types of dietary fiber. However, the traditional small-scale feeding pattern of geese, which primarily relies on gazing, is insufficient to meet the demand for goose meat. As a result, the feeding system for geese has shifted from gazing to feedlot operations [3]. Nevertheless, geese that are not fed with forage or roughage exhibit a decrease in the nutritional value of their meat compared to those supplemented with a moderate level of crude fiber [4]. Therefore, it is crucial to incorporate green feed, such as forage, into the intensive feeding pattern to ensure the health of geese and improve the quality of their meat.

Enhancing animal product quality is the primary focus of animal nutrition and feed science. Previous studies have shown that an optimal intake of crude fiber can contribute to improved geese growth performance and meat quality [5]. This includes regulating amino acid and fatty acid profiles in the muscle tissue of geese [6,7,8]. Therefore, many researchers have been studying the selection of suitable forage species and supplementation levels to enhance geese performance. However, the impact of adding dietary crude fiber still requires further investigation [5]. With the rapid development of husbandry in China, the shortage of high-quality forage has become a significant challenge in improving animal production quality [9]. Paper mulberry (*Broussonetia papyrifera*), a novel type of woody forage, possesses desirable characteristics such as strong stress resistance and high crude protein content (15–22% based on dry matter). Additionally, it contains abundant bioactive substances, including flavonoids [10,11]. Ensiling is commonly used for preserving fresh paper mulberry [12]. While most research on feeding paper mulberry has focused on ruminants, particularly lambs, there is limited information available on its impact on poultry [13,14,15]. Chen et al. reported that supplementing finishing pigs’ diet with 4% paper mulberry improved meat flavor by increasing the content of crude protein, amino acids, and total unsaturated fatty acids [16]. However, the effect of feeding paper mulberry on the growth and meat quality of geese has not been investigated. Therefore, this experiment aimed to assess the effects of dietary paper mulberry supplementation on the growth performance, carcass characteristics, meat sensory quality, chemical components, and breast muscle metabolites in geese.

## 2. Materials and Methods

### 2.1. Ethics Statement

All the geese used in this experiment were treated in compliance with Chinese animal welfare regulations. The protocols for this study were approved by the Animal Ethics Committee of China Agricultural University (AW82203202-5-1).

### 2.2. Animals and Experiment Design

One hundred and twenty 28-day-old healthy male Yangzhou geese with similar body weights were randomly allocated into either control (CON) or paper mulberry silage (PM) groups. The CON group was fed a basic diet, while the PM group was fed a diet with PM added. Each group contained 6 replicates with 10 geese per replicate. The pre-feeding period lasted for 7 days, followed by the feeding trial period within 42 days. The diets employed in this experiment were formulated to meet the nutritional requirements recommended by the National Research Council (NRC, 1994). The composition and nutrient levels of the basal diet are shown in Table 1. The PM utilized in this study was purchased from Fengtang Ecological Agricultural Technology Co., Ltd. (Taian, China), and the nutritional composition of PM was shown in Table S1. All geese had access to unlimited feed and water in the poultry feeding house.

### 2.3. Growth Performance and Sampling

Geese underwent fasting before and prior to the morning feeding on the 1st and 42nd day of the feeding trial period. The initial weight measurement was recorded on the 1st experimental day, while the final weight measurement was taken on the 42nd experiment day. The average daily feed intake (ADFI), average daily gain (ADG), and feed-to-gain ratio (F/G) were calculated by recording the body weight, feed intake, and leftover feed of geese.

On the final day of the feeding trial period, one goose in each replicate with the mean body weight of the replicate was selected and euthanized by electrical stunning and jugular vein bloodletting after fasting for 6 h. The geese carcass weight was measured after the feathers were removed. Then, the thorax was immediately opened to remove the viscera, and the eviscerated carcasses were weighed. The entire pectoral muscle was isolated and weighed post-excision of surplus adipose tissue. The mass index of carcass weight, eviscerated carcass, and breast muscle were then calculated according to the proper equation formulas [17]. The entire right breast muscles were collected to determine meat quality at 4 °C and then kept at −20 °C for analyzing the chemical composition of the geese’s meat. The left breast muscles were sampled and quickly frozen in liquid nitrogen, then stored at −80 °C until further analysis.

### 2.4. Meat Quality Determination

The water holding capacity (WHC) was determined according to the method described by Faustman [18]. The goose breast muscle sample of a 1 cm thick slice from goose breast muscle was taken for weighing. After pressurizing the steel ring of the soil permissible expansion and compression meter (B6101, Soil Instruments, Nanjing, China) to the maximum value three times and then decompressing it to zero on the micrometer, the meat sample was wrapped in gauze, placed on filter papers, and pressurized to 35 kg for 5 min on a soil allowable expansion and compression meter platform. After applying pressure, the meat was removed from the gauze and weighed promptly. Afterwards, the water loss rate under pressure was calculated. 

After the breast muscle sample was stored for 24 h at 4 °C, the pH and meat color values were measured via pH meter (PHBJ-260, Inesa Instruments, Shanghai, China) and 3NH Instruments colorimeter (NR20XE, Shenzhen, China), respectively. The lightness (L*), redness (a*), and yellowness (b*) were determined by the instrument under viewing geometry (45°/0°), measuring the aperture (Φ20 mm) and light source (D65) at room temperature and under normal light conditions. The pH and color values were measured in triplicate for each sample to obtain an average as the final values.

An official methods of analysis (AOAC) was used for determining the chemical composition [19]. The dry matter content was determined by using a vacuum method to freeze the samples at −50 °C for 5 days. Kjeldahl nitrogen was used to determine the content of crude protein, while Soxhlet extraction was used to determine the content of intramuscular fat. The crude ash was determined using the high-temperature burning method.

### 2.5. Analysis of Metabolomics 

#### 2.5.1. Extraction of Metabolites

The metabolites were extracted using previously established methods and modified as needed [20]. Accurately weighed 100 ± 2 mg meat samples were transferred into a 2 mL centrifuge tube containing 1000 μL tissue extract. After 3 steel balls were added, the samples were placed in a tissue grinder machine (50 Hz, 60 s) (MB-96, Meibi Instrument Co., Ltd., Jiaxing, China) and repeated twice. The sample was subjected to ultrasound at room temperature for 30 min followed by an ice bath for 30 min. Afterward, it was centrifuged for 10 min at 12,000 rpm and 4 °C, and the supernatant was carefully transferred into a new 2 mL centrifuge tube. The sample was concentrated and dried. To redissolve the sample, a 50% acetonitrile solution was accurately added (200 µL) and prepared with 2-chloro-l-phenylalanine (4 ppm), which was stored at 4 °C. The supernatant was then filtered with a 0.22 μm membrane and transferred into a detection bottle for LC-MS detection [21].

#### 2.5.2. LC-MS Analysis

Liquid chromatography conditions: the LC analysis was performed on an ultra-high-performance liquid phase system (Ultimate 3000, Thermo Fisher Scientific, Waltham, MA, USA). Chromatography was carried out with an ACQUITY UPLC^®^ HSS T3 (150 × 2.1 mm, 1.8 µm) (Waters, Milford, MA, USA). The column was maintained at 40 ℃. The flow rate and injection volume were set at 0.25 mL/min and 2 μL, respectively. For LC-ESI (+)-MS analysis, the mobile phases consisted of (A) 0.1% formic acid in acetonitrile (*v*/*v*) and (B) 0.1% formic acid in water (*v*/*v*). For LC-ESI (-)-MS analysis, the analytes were carried out with (C) acetonitrile and (D) ammonium formate (5 mM). Mass spectrum conditions: electrospray ion source (ESI), positive and negative ion ionization mode, positive ion spray voltage of 3.50 kV, negative ion spray voltage of 2.50 kV, sheath gas of 30 Arb, and auxiliary gas of 10 Arb. The capillary temperature was 325 °C, and the scanning range was 81~1000 with a resolution of 70,000. HCD was used for secondary cracking with a collision voltage of 30 eV, and the unnecessary MS/MS information was removed via dynamic elimination [22,23].

To effectively analyze the data, it was first converted to mzXML format using MSConvert, which is a component of the ProteoWizard software package (v3.0.8789). Next, we employed R software XCMS package (v4.1.3) to detect features, correct retention time, and align the data. To identify the metabolites, data was carefully scrutinized by considering the accuracy of the mass (ensuring it was less than 30 ppm) and cross-referenced the MS/MS data with a variety of online metabolite databases, including the Human Metabolome Database (HMDB), massbank, LipidMaps, mzcloud, and the Kyoto Encyclopedia of Genes and Genomes KEGG [24]. The data were normalized using QC-RLSC signal correction to correct for bias. Only ion peaks with RSDs less than 30% in QC were kept for metabolite identification.

#### 2.5.3. Multivariate Statistical Analysis

To analyze the impact of PM treatment on goose meat metabolites, a multivariate statistical analysis was conducted. Orthogonal projection to latent structures–discriminatory analysis (OPLS-DA) was used to evaluate the key metabolites via SMICA 14.1 (UMETRICS, Umeå, Sweden). The metabolites with a VIP greater than 1 from OPLS-DA, fold change (FC) > 2.0 or <0.5 and *p* < 0.05 were regarded as significantly differential metabolites. The differential metabolites and their respective metabolic pathways were analyzed using MetPA, which is part of Metaboanalyst 5.0 (www.metaboanalyst.ca assessed on 15 January 2021) and is mainly based on the KEGG metabolic pathway.

### 2.6. Statistic Analysis

All data were statistically processed with one-way ANOVA using SPSS 26.0 (SPSS Inc., Chicago, IL, USA). Results were displayed as mean and standard error of the mean (SEM). Values of *p* < 0.05 were considered to be statistically significant.

## 3. Results

### 3.1. Growth Performance and Carcass Characteristics

The effect of PM supplement on the growth performance and carcass characteristics of geese was shown in Table 2. Following a 7-day adaptation period, the initial body weight did not show significant difference between CON and PM groups (*p* > 0.05). The dietary PM supplementation had no significant impact on the final body weight compared with CON treatment, as well as ADFI and F/G (*p* > 0.05). And no differences were observed in the dressing percentage, eviscerated percentage, and breast muscle percentage between CON and PM groups (*p* > 0.05). However, a trend (*p* = 0.056) was observed for increased ADG (4.81%) for the supplementation with PM.

### 3.2. Meat Quality Parameters

The satisfaction of consumers with meat is greatly influenced by its sensory and nutritional qualities. As shown in Table 3, there is no significant difference in WHC and pH_24h_ of the breast muscles between the PM group and the CON group (*p* > 0.05). When considering the color of the meat, there were no significant differences in the L* and b* values between the CON and PM groups (*p* > 0.05). However, the a* value of the PM group was notably lower than that of the CON group (*p* < 0.05).

The PM group had a higher content of crude protein compared to the CON group (*p* < 0.05). No significant differences were observed in dry matter, ether extract, and ash between the two groups (*p* > 0.05).

### 3.3. Untargeted Metabolomic

#### 3.3.1. General Information for Metabolomics Data

**Figure 1:**
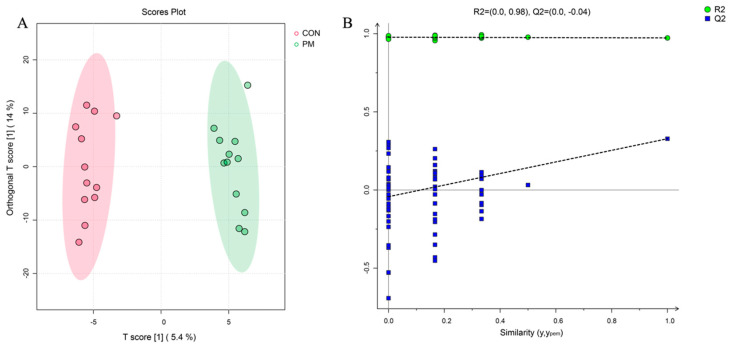
Multivariate analysis of metabolites in breast muscle of CON and PM groups. (**A**) Orthogonal projection to latent structures–discriminatory analysis (OPLS-DA) score plot. (**B**) statistical validation of OPLS-DA via 7× cross-validation and 200× permutation testing of metabolites.

The data for metabolomics were obtained using an internal mass spectrometry-based method. After analyzing the metabolites of MS/MS, a total of 572 metabolites were found across all the samples, with no missing observations. Out of these, 381 compounds were identified for metabolomics in positive ionization mode, while 191 were identified in negative ionization mode (Tables S2 and S3 provide more information). The application of OPLS-DA reduced the complexity of the model and improved its explanatory capacity, while maintaining its predictive ability. As shown in Figure 1, this analysis was also conducted on metabolites, and the validity of the permutation analysis was confirmed.

#### 3.3.2. Different Metabolites of Breast Muscle between PM and CON Groups

**Figure 2:**
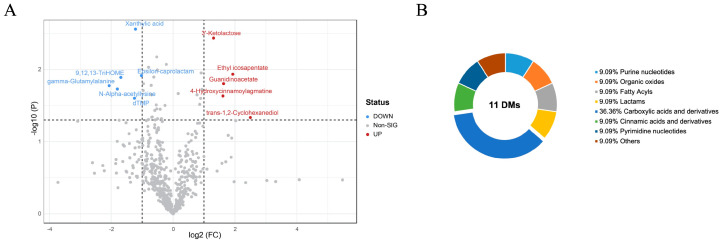
Identification of differential metabolites between CON and PM groups. (**A**) Volcano plot for differential metabolites. (**B**) Biochemical categories of identified differential metabolites are shown in a pie chart.

To identify potential biomarkers among the metabolites, the VIP obtained from the OPLS-DA was used, focusing on distinguishing the important metabolites between the PM and CON groups. A total of five metabolites were found to be upregulated, while six metabolites were downregulated, based on the established criteria of FC > 2.0 or <0.5, *p* < 0.05, and OPLS-DA VIP > 1 (Figure 2A). Further analysis revealed that these metabolites belong to various categories, including purine nucleotides, organic oxides, fatty acyls, lactams, carboxylic acids and derivatives, cinnamic acids and derivatives, pyrimidine nucleotides, and others (Figure 2B, Table 4). These metabolites significantly contribute to the observed differences between the PM and CON groups.

#### 3.3.3. Elucidating the Metabolites Difference and Pathway

**Figure 3:**
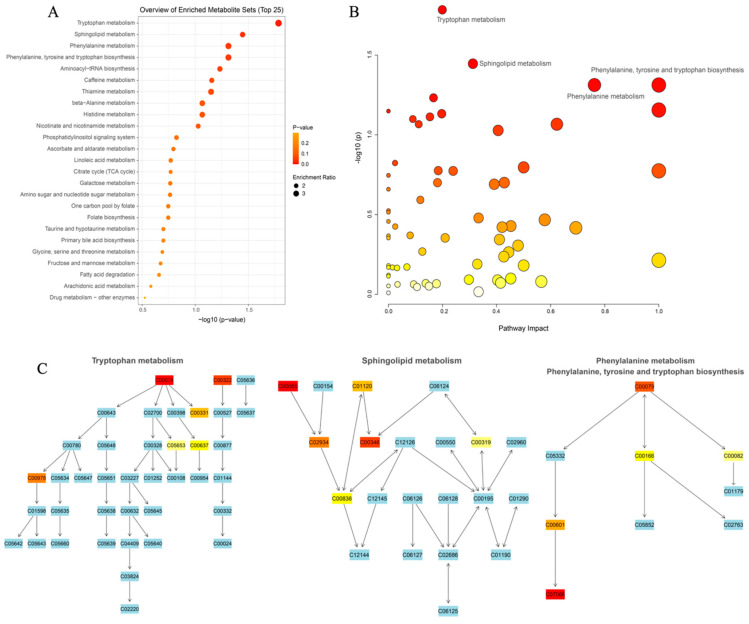
Function analysis for the metabolites. (**A**) Metabolite set enrichment analysis. (**B**) KEGG pathway analysis. (**C**) The significantly enriched KEGG pathway is involved via different metabolites (in the red background).

In order to compensate for the limited significant metabolites between the PM and CON groups, MS/MS metabolite data was utilized for MSEA and KEGG pathway analysis with the help of Metaboanalyst. The findings from the metabolite set enrichment analysis (MSEA) revealed significant enrichment in tryptophan metabolism, sphingolipid metabolism, phenylalanine metabolism, and phenylalanine, tyrosine, and tryptophan biosynthesis (*p* < 0.05) (Figure 3A). The study revealed that the metabolites L-tryptophan and 2-oxoadipate were found to be enriched in tryptophan metabolism. Additionally, L-serine and ethanolamine were enriched in sphingolipid metabolism, while phenylacetic acid was enriched in phenylalanine metabolism. Interestingly, L-phenylalanine was found to be enriched in both phenylalanine metabolism and phenylalanine, tyrosine, and tryptophan biosynthesis (*p* < 0.05). These findings emphasize the close association between amino acid metabolism and biosynthesis, as depicted in Figure 3B,C and Table S5.

## 4. Discussion

### 4.1. Growth Performance, Carcass Characteristics, and Meat Quality of Yangzhou Geese

Geese are herbivorous poultry capable of efficiently digesting and utilizing green forage. The addition of PM to their diet can meet their grazing needs while increasing dietary fiber intake, which enhances their health and well-being, ultimately reducing the risk of diseases and undesirable behaviors [25]. There was no significant effect on the body weight between the PM group and the CON group at 28 days of age; however, the PM group’s weight was slightly lower than that of the CON group. This may be attributed to the dietary adjustment in the PM group during the pre-test period.

The impact of the diet on the growth performance of geese can be assessed through parameters such as the ADFI and ADG. Liu et al. showed that with an increase in dietary crude fiber levels, geese exhibited an upward trend in ADFI, and a pattern of initial increase followed by a decrease in ADG [26]. However, it significantly impacted the ADFI, with the geese’s feed intake gradually decreasing as the proportion of paper mulberry leaf powder increased. In this experiment, there was no significant effect on the ADFI between the PM group and the CON group. Nevertheless, the addition of PM had a promoting effect on the ADG of geese, which demonstrates the good quality of PM.

The carcass characteristics, such as dressing percentage and eviscerated percentage, serve as reliable indicators of an animal’s nutrient intake from the diet. Among these characteristics, the quality of breast muscle plays a crucial role in determining poultry meat production performance [27]. Aslan et al. demonstrated that the inclusion of corn silage in geese diets did not significantly impact their dressing percentage [28]. In line with previous studies showing higher breast muscle percentages in geese that were fed a combination of grass and grain compared to those on a single diet, our study found that supplementing with PM also increased breast meat percentage [29]. Desbuards et al. demonstrated that augmenting the dietary fiber content in the maternal diet during pregnancy and lactation resulted in amplified colon length and body weight in the offspring, particularly an increase in leg muscle weight [30]. It is postulated that this alteration is associated with the absorption of dietary fiber by the gut microbiota, encompassing the secretion of hormones such as insulin and growth hormone, which are acknowledged to stimulate protein synthesis and foster muscle development. Consequently, it can be inferred that the elevation in breast muscle percentage observed within our experiment’s PM group may be attributed to incorporating paper mulberry into their diet, thereby leading to a heightened intake of fiber by geese.

The assessment of meat quality encompasses both sensory and nutritional aspects. Sensory quality primarily comprises pH value, water holding capacity, and meat color. The pH level directly reflects muscle acidity and significantly influences the heat resistance, cooking loss, and processing performance of muscles. An elevation in pH levels leads to a reduction in the rate of anaerobic glycolysis of muscle glycogen, thereby minimizing water exudation loss, promoting greater protein structure stability, and enhancing overall meat freshness [31]. In this experiment, both the PM group and the CON group exhibited similar pH values within the normal range (5.8–6.2). Water holding capacity can affect the edible quality of meat, including its color, aroma, and taste, etc. Xiong et al. showed that adding a certain amount of PM to the diet has no effect on the pH of goat meat and reduces dripping loss in goat meat, while our results indicated that there is no significant difference in water holding capacity of the breast muscles between the PM group and the CON group [9]. This may be related to the different muscle compositions among various species. Meat color is influenced by factors such as myoglobin content, residual hemoglobin content, myohemoglobin amount, oxidation state, and surface light reflection ability [32]. Goose meat is classified as white meat, and a slightly pinkish hue can promote consumer purchases. In this experiment, the a* value, which represents the redness of meat, was lower in the PM group; this indicated that the addition of PM to the diet has a positive impact on the color of goose breast meat. The nutritional value of goose meat is influenced by the levels of chemical components like protein and fat, and the higher the protein content, the better the nutritional value of meat. In this experiment, the addition of PM to the diet improved the nutritional quality of goose breast meat, with significantly higher protein content in the PM group compared to the CON group. Liu et al. showed that the effect of alfalfa-mixed silage can increase muscle protein content and decrease the crude fat content of geese meat [33]. This may be related to the presence of a significant amount of digestible protein and an appropriate amino acid composition in the forage, thereby promoting the deposition of protein in goose meat. Overall, the use of PM feed can improve the sensory and nutritional quality of goose meat to some extent.

### 4.2. Untargeted Metabolomic (LC-MS/MS) Analysis of Breast Muscle in the CON and PM Groups

Metabolomics is employed for the rapid screening of small molecule metabolites in tissues, fluids, and cells under specific conditions. The dietary factor plays a pivotal role in determining the quality of raw meat. Numerous animal studies utilize metabolomics to evaluate the dietary value of raw meat. Although direct comparisons between individual experiments are not feasible due to inherent variations in methodologies and experimental setups, these findings can serve as valuable references for future investigations [34]. To characterize the alterations in metabolites between the CON and PM groups of breast muscle, LC/MS-MS was employed for the identification of metabolite composition and content. Subsequently, significant differential components were screened and categorized into eight distinct classes. Notably, among these categories, five substances exhibited upregulation while six substances displayed downregulation. To ensure accuracy and consistency with previous studies [35,36], guanidinoacetate and 9,12,13-TriHOM were selected as marker metabolites for upregulation and downregulation, respectively.

Compared to the CON group, the expression of guanidinoacetic acid is upregulated in the paper mulberry PM group. Guanidinoacetic acid is an endogenous precursor of creatine, produced from amino acids and their derivatives. It should be noted that plant-based foods lack creatine and it is primarily obtained from various animal-derived sources or synthesized endogenously within the body [37,38]. This finding suggests that incorporating PM into geese feed can enhance amino acid absorption and promote creatine synthesis in muscle tissue. As a valuable feed additive, it has potential applications in promoting animal fattening and improving meat’s nutritional quality [39]. Moreover, Reicher et al. demonstrated that supplementing guanidinoacetic acid in broiler feed enhances nutrient absorption and deposition into breeding eggs, which may have implications for guanidinoacetic acid absorption and creatine synthesis in broiler progeny [40].

Compared to the CON group, the expression of 9,12,13-TriHOM is downregulated in the PM group. It was the metabolite of linoleic acid, vascular tissue converted various polyunsaturated fatty acid into monohydroxy, and trihydroxy metabolites derived from hydroperoxide, esterified oxygenated polyunsaturated fatty acids incorporated into lipids, which could not be well incorporated into vascular endothelium or smooth muscle cells, or it can accumulate in lipids due to autooxidation or enzymes similar to lipoxygenase in the body [41,42]. The antioxidant properties of meat products are directly related to their sensory qualities and are indirectly reflected in changes in protein functionality and amino acid loss [43]. Dietary plant-based additives can be used to combat lipid oxidation. Mattioli et al. showed that the use of herbs rich in flavonoid compounds as dietary additives in rabbit feed can enhance the antioxidant capacity of rabbit meat [44]. Orlowski et al. showed that phytogenic additives in the diet may improve chicken meat quality by modulating stress and antioxidant-related pathways [45]. Wang et al. showed that the dietary supplementation of pterostilbene may enhance the activities of total superoxide dismutase, catalase, and glutathione peroxidase while reducing lipid and protein oxidation, and this leads to an improvement in the sensory characteristics and nutritional value of chicken meat [46]. Therefore, we speculate that after consuming the diet with PM, geese undergo a series of complex digestive and absorption processes, and these processes may have varying effects on metabolic pathways involving amino acids and lipid oxidation. The combined effects of these processes enhance the sensory quality and nutritional value of goose meat, and this hypothesis may be related to the changes observed in the chest muscle indicators mentioned earlier, such as the breast–muscle ratio and its protein content; further research is needed to explore the underlying mechanisms.

Further analysis of metabolic pathways was conducted. However, due to the limited number of differentially detected metabolites, pathway enrichment analysis was performed on all substances, leading to the identification of four significantly enriched metabolic pathways. Notably, tryptophan metabolism emerged as the most significant enrichment pathway. Tryptophan is an indispensable amino acid in poultry and serves as a precursor for numerous vital molecules within the body. Moreover, it is directly or indirectly regulated by intestinal microorganisms [47]. Sphingolipid metabolism appeared as the second significant metabolic pathway and exhibits interconnectedness with fatty acid metabolism through key enzymes such as SPT, serine, and fatty acyl-CoA [48]. The synthesis and metabolism of phenylalanine, which represents the final significant metabolic pathway to emerge, involves its oxidation to tyrosine through the catalytic action of phenylalanine hydroxylase. Tyrosine, in turn, plays a crucial role in neurotransmitter and hormone synthesis while also participating in glucose and lipid metabolism [49]. These findings suggest that the inclusion of PM may exert an influence on amino acid and lipid metabolism in geese. As previously hypothesized, the significant differential metabolites, guanidinoacetic and 9,12,13-TriHOM, appear to be associated with the amino acid and lipid pathways in geese. The predicted pathways align well with these findings. Furthermore, LC-MS/MS analysis provides evidence that guanidinoacetic is integrated into the metabolic pathways of glycine, serine, and arginine as a precursor to creatine (Figure S1). 

### 4.3. Flavor Compounds Analysis of Breast Muscle in the CON and PM Groups

In the determination of nutritional quality, we found that the protein content of the PM group was higher than that of the CON group. Amino acid is the basic unit of protein, and it is also an essential metric for assessing the flavor of meat, and thus altering the amino acid composition to alter the flavor of meat creates a wide range of possibilities [50,51]. The primary umami amino acids are L-aspartic acid and glutamic acid, while the primary sweet amino acids are glycine and alanine. After metabolite data were analyzed (Tables S2 and S3), it was discovered that the PM group had a higher L-aspartate acid and glutamic acid level than the CON group; glycine and alanine were not directly found in this experiment. This showed that PM may improve the umami taste of goose meat. We also found that the content of the intramuscular fat of the PM group was slightly lower than that of the CON group. The primary components of intramuscular fat are fatty acids: its amount and makeup of these molecules have a significant impact on the flavor and quality of meat [52]. Aldehydes, ketones, and acidic chemicals are produced during the oxidation of muscle-derived fatty acids, which are primarily polyunsaturated (PUFA) and monounsaturated (MFA) [53]. According to Tables S2 and S3, the MFA and PUFA substances in the PM group were marginally lower than those in the CON group during this experiment, such as linolic acid and α-linolenic acid; during hot processing, they released large amounts of aldehydes, which give meat a rich, volatile flavor. This showed that PM may have a certain impact on the flavor of Yangzhou goose breast muscle. We speculated that this might be because the PM and CON diets differ in terms of lipid composition, which would alter how the goose body transports fat and energy.

Additionally, LC-MS/MS-based data identified a number of other flavor compounds, and some of them are listed separately in Table S4, such as valeric acid, isovaleric acid, heptanoic acid, phenylacetaldehyde, m-Cresol, phenylacetic acid, 2,3-butanediol, and benzaldehyde. With the exception of m-Cresol, these substances showed a downward trend. Valeric acid, isovaleric acid, and heptanoic acid have sweetness characteristics [54]. Phenylacetaldehyde dehydrogenase catalyzes the NAD + -dependent oxidation of phenylacetaldehyde to phenylacetic acid; both of them have a strong honey taste [55,56]. 2,3-butanediol is similar to a butter smell, while benzaldehyde has a distinctive almond-like smell and taste [57,58]. This indicated that the taste of sweetness of goose breast muscle in the PM group was lower than that in the CON group. M-Cresol is one of the key volatile phenolic components in beef fat [59], and it is also one of the key factors in beef flavor [60], suggesting that PM may promote the unique flavor of goose breast. The above flavor compounds were rarely reported in goose breast; no straightforward reasons could explain such observations. It is therefore suggested that more complex interactions and mechanisms could be involved in the formation of specific key odor-active compounds [61].

Feed plays an important role in imparting certain flavor characteristics in poultry [62]. In future studies, it would be advantageous to consider flavor omics and integrating proteomics methods to investigate the changes in the flavor quality of goose meat. Additionally, exploring whether these favorable changes can be implemented in production will address consumers’ demands for high-quality, safe, and nutritious goose meat products. This research holds great importance in meeting such requirements while maintaining scientific rigor within the field.

## 5. Conclusions

In conclusion, this study demonstrates that the inclusion of paper mulberry silage in the diet has a promotional effect on the growth of Yangzhou geese. Moreover, it promotes breast muscle development and significantly enhances meat quality. Additionally, it increases the protein content in meat, elevating levels of substances like guanidinoacetic acid in the breast muscles of Yangzhou geese, while substances like 9,12,13-TriHOM exhibited decreased levels, and these changes contribute to the enhancement of meat quality and antioxidant capacity in Yangzhou geese. All these findings provide valuable insights into understanding how paper mulberry affects growth, development, and meat quality in Yangzhou geese. In the future, a focus should be placed on exploring the impact of substances within paper mulberry on goose meat.

## Figures and Tables

**Table 1:** Composition and nutrient level of experiment diets (air-dry basis) (%).

Item	Groups	
	CON	PM
Ingredient		
Corn	60.00	57.30
Wheat bran	15.50	12.50
Soybean meal	20.50	18.25
Paper mulberry silage	0.00	8.00
CaHPO_4_	0.20	0.20
Shell	0.15	0.05
NaCl	0.30	0.30
Met	0.15	0.20
Premix	3.20	3.20
Total	100.00	100.00
Nutrient level		
ME/(MJ·kg^−1^)	12.21	12.15
CP	16.50	16.50
CF	3.82	5.20
Ca	0.76	0.77
P	0.57	0.54
Lys	0.78	0.70
Met	0.77	0.76

The premix provided the following per kilogram of diets VA 6700 IU, VD3 1500 IU, VE 14 mg, VK3 1.75 mg, VB1 1.20 mg, VB2 4.35 mg, VB6 2.85 mg, VB12 0.002 mg, nicotinic 29.00 mg, D-calcium pantothenate 7.85 mg, folic acid 7.85 mg, D-biotin 0.08 mg, choline 3.00 mg, Ca 550 mg, P 370 mg, Fe 60 mg, Cu 5 mg, Mn 6.5 mg, Zn 60 mg, I 3.5 mg, and Se 0.25 mg; nutrient levels are calculated values.

**Table 2:** Effects of CON and PM diet supplementation on initial body weight, final body weight, average daily gain, dressing percentage, eviscerated percentage, and breast muscle percentage.

Item	Groups		SEM	*p*-Value
	CON	PM		
Live weight, g				
D_1_	632.00	612.89	8.65	0.421
D_42_	3168.5	3277.56	34.17	0.095
ADFI, g	323.64	338.65	8.35	0.690
ADG, g	60.39	63.44	0.79	0.056
F/G	5.58	5.60	0.08	0.841
Dressing percentage, %	84.15	83.12	0.51	0.343
Eviscerated percentage, %	68.06	68.50	0.65	1.000
Breast muscle percentage, %	7.65	8.08	0.17	0.486

D_1_, the initial live weight of the geese (28-day-old). D_42_, the final live weight of the geese (70-day-old).

**Table 3:** Effects of CON and PM diet supplementation on breast meat quality.

Item	Groups		SEM	*p*-Value
	CON	PM		
WHC, %	28.25	27.81	0.61	0.765
pH_24h_	5.85	5.87	0.02	0.629
Meat color				
L*	49.59	47.77	0.62	0.157
a*	22.36	19.69 **	0.63	0.005
b*	10.44	10.63	0.10	0.407
Dry matter, %	22.07	23.55	0.44	0.089
Crude protein, %	79.35	80.42 *	0.28	0.041
Intramuscular fat, %	9.05	8.91	0.11	0.588
Ash, %	1.53	1.55	0.03	0.728

Calculation of crude protein, ether extract, and ash based on dry matter, * *p* < 0.05 and ** *p* < 0.01, compared with the CON group.

**Table 4:** The detailed results of differential metabolites in the geese breast muscle (OPLS-DA VIP > 1, FC > 2 or <0.5, and *p*-value < 0.05).

Name	Adduct	*m*/*z*	rt(s)	VIP	FC	Variation	*p*-Value
Xanthylic acid	[M − H]^−^	363.0339	74.2	2.5624	0.43003	↓	0.0027389
3′-Ketolactose	[M − H]^−^	339.0944	345.9	2.4382	2.47560	↑	0.0036448
Ethyl icosapentate	[M + H]^+^	331.2631	846.6	1.9362	3.81320	↑	0.0115820
Epsilon-caprolactam	[M + H]^+^	114.0918	423.6	1.9165	0.49254	↓	0.0121200
9,12,13-TriHOME	[M − H]^−^	329.2334	642.8	1.8918	0.31053	↓	0.0128290
Guanidinoacetate	[M − H]^−^	116.0494	366.1	1.8054	3.10280	↑	0.0156530
gamma-Glutamylalanine	[M + H]^+^	219.0952	774.9	1.7771	0.23896	↓	0.0167070
N-Alpha-acetyllysine	[M]^+^	118.0710	399.1	1.7316	0.28660	↓	0.0185530
4-Hydroxycinnamoylagmatine	[M]^+^	276.1447	237.4	1.6351	3.05340	↑	0.0231710
dTMP	[M − H]^−^	321.0482	123.1	1.6030	0.42068	↓	0.0249450
trans-1,2-Cyclohexanediol	[M]^−^	115.9194	931.1	1.3355	5.64660	↑	0.0461810

↓ means down-regulation, and ↑ means up-regulation, compared with the CON group.

## Data Availability

The data presented in this study are available on request from the author. The data are not publicly available due to the privacy policy of the institute.

## Supplementary Materials

The following supporting information can be downloaded at: https://www.mdpi.com/article/10.3390/ani14030359/s1, Figure S1: Visualization of creatine pathway and glycine, serine, and arginine metabolism are associated with a network; Table S1: Nutrient level of paper mulberry silage (air-dry basis); Table S2: Identified for 381 metabolomics in positive ionization mode; Table S3: Identified for 191 metabolomics in negative ionization mode; Table S4: The details of some flavor compounds of breast muscle in the CON and PM groups; Table S5: The details of the significantly enriched KEGG pathway are involved by different metabolites (in the red background).
