# Supplementary material for: Dietary Paper Mulberry Silage Supplementation Improves the Growth Performance, Carcass Characteristics, and Meat Quality of Yangzhou Goose
Source: Animals (Basel). 2024 Jan 23;14(3):359. doi: 10.3390/ani14030359 (PMC10854908; doi:10.3390/ani14030359)

Dietary Paper Mulberry Silage Supplementation Improves the Growth Performance, Carcass Characteristics, and Meat Quality of Yangzhou Goose

Ruhui Wang [1,2], Xin Wang [1], Yi Xiong [1], Jingwen Cao [1], Luiz Gustavo Nussio [3], Kuikui Ni [1], Yanli Lin [1], Xuekai Wang [1,*] and Fuyu Yang [1,2,*]

[1] College of Grassland Science and Technology, China Agricultural University, Beijing 100193, China; wangruhui9288@163.com (R.W.); wangxin1814@126.com (X.W.); caojingwen1207@163.com (J.C.)
[2] College of Animal Science, Guizhou University, Guiyang 550025, China
[3] Department of Animal Sciences, Luiz de Queiroz College of Agriculture, University of Sao Paulo, Piracicaba 13418-900, Brazil; nussio@usp.br
* Correspondence: xkwang@cau.edu.cn (X.W.); yfuyu@126.com (F.Y.); Tel.: +86-010-62733-052 (F.Y.)

Supplementary Materials

Table S1 Nutrient level of paper mulberry silage (air-dry basis). %

Item	Content	Item	Content	Item	Content
Moisture	64.30	ADF	25.70	Ca	1.49
Dry Matter	35.70	NDF	30.30	P	0.28
CP	20.10	Ash	7.40	Mg	0.47
EE	4.90	WSC	10.20	K	1.60

Table S2 Identified for 381 metabolomics in positive ionization mode.

No.	Name	m/z	rt(s)	Addut	CON	PM	Variation
1	D-Lysopine	201.1242	354.4	[M+H-H2O]+	2517824.34	7528566.11	↑
2	S-(Hydroxymethyl)glutathione	320.0911	169.7	[M+H-H2O]+	19288442.3	12387832.2	↓
3	CDP-Ethanolamine	447.0667	89.5	[M+H]+	7522634.13	12535327.1	↑
4	Etiocholanedione	289.2163	842.8	[M+H]+	59214247	10177037	↓
5	Phenylacetaldehyde	121.0648	340	[M+H]+	9949676.92	5971588.47	↓
6	Tyramine	138.0916	337.2	[M+H]+	2887111.59	3329489.31	↑
7	Norlinolenic acid	264.2064	463.4	[M]+	545483.128	547488.846	↑
8	Gentamicin X2	482.2621	967.2	[M]+	46884716.1	12958882.9	↓
9	Cucurbitacin B	558.3189	905.6	[M]+	7177503.21	6933842.96	↓
10	Cucurbitacin A	574.3139	822.8	[M]+	7208166.96	9019055.6	↑
11	L-Olivosyl-oleandolide	516.3047	813.5	[M]+	27299765.6	18222887.4	↓
12	Methylmalonic acid	101.0711	934.7	[M+H-H2O]+	22404759.6	23589052.5	↑
13	Triethylamine	102.128	32.5	[M+H]+	26092386.2	23830689.4	↓
14	Valeric acid	102.0911	533.4	[M]+	3326764.92	2965388.44	↓
15	Isovaleric acid	102.0912	377.7	[M]+	5235997.17	4379589.7	↓
16	gamma-Aminobutyric acid	104.1074	85.7	[M+H]+	777526992	715965414	↓
17	m-Cresol	109.0653	188.6	[M+H]+	8526141.64	8971680.77	↑
18	Hydroquinone	110.0202	977.8	[M]+	102547279	79127987.6	↓
19	Pyrrole-2-carboxylic acid	111.021	877	[M]+	118248556	98328717.5	↓
20	Uracil	113.0349	125.2	[M+H]+	114921937	95799751	↓
21	Cytosine	112.0514	100.7	[M+H]+	86429498.2	72459345.6	↓
22	Quinoline	112.0506	148.4	[M+H-H2O]+	67014075.2	18674340.4	↓
23	Creatinine	113.9641	143.6	[M+H]+	157956418	278550692	↑
24	3-Methylindole	132.081	805.6	[M+H]+	8250887.59	7634526.33	↓
25	L-Proline	116.0707	97.7	[M+H]+	1324591276	933280424	↓
26	Betaine	118.0862	90.8	[M+H]+	737871141	426725761	↓
27	Indole	117.0578	399.7	[M]+	258791699	240176197	↓
28	L-Threonine	120.0659	88	[M+H]+	306295582	178600937	↓

29	Phenylacetic acid	136.0495	80.6	[M]+	9337718.64	6480346.53	↓
30	4-Hydroxystyrene	121.0287	829.2	[M+H]+	31984385.2	26986097.3	↓
31	Tyrosol	121.0651	189.3	[M+H-H2O]+	3707640.35	4804729.64	↑
32	L-Cysteine	122.0275	485	[M+H]+	1384287.62	5145632.75	↑
33	Niacinamide	123.0548	237.3	[M+H]+	88606341.9	126959471	↑
34	Thymine	127.0505	338.3	[M+H]+	14457171.7	11139300.1	↓
35	Pipecolic acid	130.05	86.9	[M+H]+	2019170625	2326254515	↑
36	Heptanoic acid	130.9673	254.6	[M+H]+	12088932.6	7431762.03	↓
37	cis-4-Hydroxy-L-proline	130.9673	205.6	[M]+	4513894.49	2852493.87	↓
38	trans-Cinnamate	131.0495	340.1	[M+H-H2O]+	111178243	105056905	↓
39	Nornicotine	149.0602	308.9	[M+H]+	503131.141	980879.812	↑
40	(R)-Pantolactone	131.5337	977.8	[M+H]+	16377421.7	9487051.81	↓
41	Glutaric acid	131.9744	60.1	[M]+	103043324	102470249	↓
42	L-Leucine	132.102	217.8	[M+H]+	1616000501	1509337015	↓
43	L-Isoleucine	132.1019	177.6	[M+H]+	901167876	887855968	↓
44	L-Asparagine	133.1057	157.5	[M+H]+	87021378.6	98621182.9	↑
45	Xanthine	153.0402	164.1	[M+H]+	752665089	618899865	↓
46	2-Hydroxyphenethylamine	137.0792	188.9	[M]+	25821955.5	22425795.8	↓
47	Uracil 5-carboxylate	156.1206	779.6	[M]+	9398324.23	4809130.42	↓
48	Urocanic acid	139.0506	284	[M+H]+	73577700.9	63274912.6	↓
49	Phosphonoacetate	140.0709	118.9	[M]+	8138674.23	9598163.25	↑
50	Allantoin	158.9617	62.8	[M+H]+	66455612.7	44404964.7	↓
51	Tryptophanol	144.0804	399.1	[M+H-H2O]+	704645152	579852304	↓
52	L-Carnitine	162.112	89.4	[M+H]+	5742485730	5227456899	↓
53	Anabasine	144.9819	793.6	[M+H-H2O]+	46089133.7	42797180.8	↓
54	3-Hydroxymethylglutaric acid	144.9818	892.7	[M+H-H2O]+	728909250	495424101	↓
55	L-Lysine	146.0604	338.6	[M]+	1470607.97	1790587.85	↑
56	L-Glutamic acid	148.0606	89.3	[M+H]+	659551695	676514734	↑
57	L-Glutamine	147.0765	86.9	[M+H]+	1195821995	1323116956	↑
58	L-2-Hydroxyglutaric acid	148.9773	60.6	[M+H]+	36176913.3	27980891.9	↓
59	3-Methyladenine	149.0231	459	[M]+	25580393.8	25286627.4	↓
60	L-Methionine	150.0589	125.1	[M+H]+	589282280	711821198	↑
61	Pyridoxine	152.071	99.3	[M+H-H2O]+	10513257.6	8201462.5	↓
62	1-Methylhistidine	170.0923	225.1	[M+H]+	8777634.15	3330493.29	↓
63	3-Hydroxyphenylacetic acid	153.0112	987.6	[M+H]+	28373427.2	32394262.8	↑
64	Mandelic acid	152.9947	60.6	[M+H]+	16500259.6	10919871.6	↓
65	3-Amino-4-hydroxybenzoate	153.9933	59.2	[M+H]+	2649911.26	2390077.24	↓
66	2,3-Butanediol	154.0444	319.5	[M]+	8031221.35	4376043.89	↓
67	Gentisic acid	154.9898	977.8	[M+H]+	788762678	701619011	↓
68	3-Dehydroshikimate	171.9931	332.3	[M]+	19390296	12297978.2	↓
69	4-Quinolinecarboxylic acid	155.9928	987.6	[M+H-H2O]+	62882794.2	87410179.6	↑
70	L-Histidine	156.0769	76.1	[M+H]+	112496871	100482037	↓
71	4,5-Dihydroorotic acid	158.9611	292.4	[M+H]+	49763668.6	35338356.9	↓
72	N-Methyl-L-glutamic acid	162.0764	99.2	[M+H]+	45155512.3	47393670.9	↑

73	Acetylcysteine	162.9981	35.5	[M]+	10703602.7	9199312.69	↓
74	Pterin	163.0388	712.3	[M]+	9837749.27	10658467.4	↑
75	L-Fucose	165.1138	973.4	[M+H]+	20896286.3	18610838.2	↓
76	6-Methylmercaptopurine	166.9883	706.4	[M+H]+	1253239.9	1503516.58	↑
77	L-Phenylalanine	166.0864	340.1	[M+H]+	3244558418	1846967845	↓
78	D-Ornithine hydrochloride	169.0613	329	[M+H]+	2552635.33	3400519.61	↑
79	Norepinephrine	169.9777	332.3	[M+H]+	67264841.7	111530271	↑
80	N-Alpha-acetyllysine	188.071	399.1	[M]+	6242123247	1789012353	↓
81	2-Aminobenzenesulfonate	174.0224	451.8	[M+H]+	8387118.39	6845998.81	↓
82	L-Arginine	175.1193	84.5	[M+H]+	612973199	656827968	↑
83	Ascorbate	176.9722	46.5	[M+H]+	4053929.07	5985494.76	↑
84	Methyl beta-D-galactoside	176.9721	60.1	[M+H-H2O]+	5384421.3	6760865.7	↑
85	D-Gulono-1,4-lactone	178.1342	400.1	[M]+	5563657.67	5795220.04	↑
86	myo-Inositol	181.0153	877	[M+H]+	122783240	92086060.5	↓
87	Homovanillic acid	183.0842	162.6	[M+H]+	66145381.9	34206898.4	↓
88	L-Tyrosine	182.0815	126.2	[M+H]+	654348800	576564231	↓
89	Sorbitol	182.9847	32.9	[M+H]+	23483151.1	15972600.2	↓
90	Mannitol	182.0817	395.9	[M]+	15135663.9	11906629.6	↓
91	Epinephrine	184.0974	359.8	[M+H]+	78847847.3	88123191.9	↑
92	Undecanoic acid	186.9566	62.4	[M+H]+	1324643.68	927010.885	↓
93	N-Acetylglutamic acid	190.0717	835.3	[M+H]+	13251521.5	19834435.7	↑
94	D-Glucuronic Acid	195.123	356.6	[M+H]+	1759399.24	1438118.18	↓
95	Vanillylmandelic acid	198.1855	692.9	[M]+	6027157.49	9206749.75	↑
96	Dodecanoic acid	199.9883	35.5	[M]+	4419171.28	4043199.43	↓
97	Spermine	203.2235	68.8	[M+H]+	43793751.4	40732423.4	↓
98	L-Tryptophan	205.0977	399.1	[M+H]+	354044019	242871918	↓
99	Butyrylcarnitine	231.2517	731.2	[M]+	2291630.89	2682806.62	↑
100	12-Hydroxydodecanoic acid	216.1962	659	[M]+	459468.899	673293.341	↑
101	Anserine	240.0981	89.2	[M]+	3153376.32	11575796.8	↑
102	Hydroxykynurenine	225.0871	115.5	[M+H]+	7375154.03	6870092.2	↓
103	Porphobilinogen	226.1805	693	[M]+	10838929.5	11251369.8	↑
104	Deoxyuridine	228.1961	747.5	[M]+	15860418.5	24820129.6	↑
105	D-Ribose 5-phosphate	230.2479	752.9	[M]+	8981579.18	13848785.1	↑
106	Lumichrome	243.0881	569.5	[M+H]+	32369727.9	10496533.8	↓
107	1-Hexadecanol	243.1828	378.7	[M+H]+	17462005	27581761.8	↑
108	Adenosine	250.094	336.5	[M+H-H2O]+	16340247.1	19773868.3	↑
109	Palmitic acid	256.2639	953.1	[M]+	17016931.8	20685546.5	↑
110	Alpha-Linolenic acid	279.232	857.9	[M+H]+	163722511	89409586.1	↓
111	Linoleic acid	280.2641	939.1	[M]+	233838752	196247197	↓
112	(6Z)-Octadecenoic acid	265.2526	821.5	[M+H-H2O]+	177759880	141881798	↓
113	5'-Methylthioadenosine	298.0977	387.3	[M+H]+	1381502098	1334130761	↓
114	Oleic acid	282.279	822.8	[M]+	944757287	986669155	↑
115	Guanosine	284.099	282.5	[M+H]+	91041237.2	58793665	↓
116	Glycitein	285.0762	629.9	[M+H]+	179463.838	140375.023	↓

117	Fisetin	286.2743	757.2	[M]+	7190030.07	6616831.9	↓
118	8,11,14-Eicosatrienoic acid	307.2638	895	[M+H]+	6932503.36	6484641.94	↓
119	Glutathione	307.0802	168.6	[M]+	2617816415	1976937810	↓
120	Aflatoxin B1	312.3627	847.3	[M]+	18379280.4	14932725.1	↓
121	Pregnanediol	321.3152	893.1	[M+H]+	126744607	170119391	↑
122	CMP	324.058	100.7	[M+H]+	82205818.2	45186448.5	↓
123	Docosahexaenoic acid	328.1996	456.6	[M]+	1279447.42	962530.689	↓
124	Nicotinamide ribotide	335.066	102.1	[M+H]+	50378129.9	55787273.9	↑
125	Erucic acid	338.3425	893.9	[M]+	4947915142	5195310352	↑
126	Sucrose	343.296	739.6	[M+H]+	4434654.98	4007425.33	↓
127	IMP	349.0549	123.4	[M+H]+	1738911200	1441860485	↓
128	Thyrotropin releasing hormone	362.3249	732.7	[M]+	7249689.92	9043241.01	↑
129	Riboflavin	377.1456	449.8	[M+H]+	33807543.7	19787566.5	↓
130	Vitamin D3	367.336	941.3	[M+H-H2O]+	15220327.3	9545544.51	↓
131	Cholesterol	387.1929	799.6	[M+H]+	6729616.36	7420462.72	↑
132	Deoxycholic acid	393.2849	798.1	[M+H]+	700833202	701492391	↑
133	Ergocalciferol	397.1987	805.6	[M+H]+	44277665.2	22549778.6	↓
134	S-Adenosylmethionine	399.1446	85.5	[M+H]+	82606848	87801524	↑
135	Allocholic acid	408.3687	903.7	[M]+	10724124.8	7576626.5	↓
136	Lanosterin	427.2748	640.6	[M+H]+	721403.176	847307.872	↑
137	alpha-Tocopherol	430.2429	532.5	[M]+	28734778.1	45275294	↑
138	Sodium deoxycholate	415.2112	805.6	[M+H]+	193397011	1577591473	↑
139	Isovitexin	432.2345	804.3	[M]+	25930831.5	36589809.2	↑
140	Deoxyuridine-5'-triphosphate	468.3902	973.1	[M+H]+	10475996.8	8966958.41	↓
141	ATP	507.2787	919	[M]+	9866866.8	9028914.51	↓
142	beta-Carotene	536.165	964.3	[M]+	57230265.1	44719930.2	↓
143	Bilirubin	584.4722	895	[M]+	23801487.8	19482362.8	↓
144	Rutin	610.1812	962.2	[M]+	40271475.2	18067203	↓
145	Maltotetraose	649.225	109.5	[M+H-H2O]+	63384028	49524823.1	↓
146	Stachyose	667.2271	102.2	[M+H]+	30739324.3	15587633.4	↓
147	Choline	104.1071	685.3	[M+H]+	1291122.58	1840852.45	↑
148	Benzaldehyde	107.0495	746.2	[M+H]+	89130279.9	70907821.4	↓
149	o-Xylene	107.0859	805.6	[M+H]+	7966680.96	6691618.15	↓
150	Succinic acid	119.0356	124.3	[M+H]+	818135407	681853692	↓
151	5-Aminoimidazole-4-carboxamide	126.056	61.1	[M]+	2574207.82	3035844.02	↑
152	1-Methylhistamine	126.1029	84.8	[M+H]+	152400070	130957522	↓
153	1,2,3-Trihydroxybenzene	127.038	103.6	[M+H]+	473529464	372251111	↓
154	Beta-Guanidinopropionic acid	132.0779	282.6	[M+H]+	52630391.1	61025415.2	↑
155	Chavicol	135.0807	630.1	[M+H]+	647768.133	880779.222	↑
156	p-Anisaldehyde	137.0593	188.4	[M+H]+	18012658.3	23797722.4	↑
157	Kojic acid	143.0341	105	[M+H]+	121821587	86241817.2	↓
158	3-(2-Hydroxyphenyl)propanoic acid	149.06	830.3	[M+H-H2O]+	3366030.94	3620658.03	↑
159	Guanine	152.057	152.3	[M+H]+	399336409	302698158	↓
160	Indoleacetaldehyde	160.0761	399	[M+H]+	40035285.3	48550131.6	↑

161	Cysteinylglycine	179.0487	124.7	[M+H]+	13262428	14590685.7	↑
162	Beta-Tyrosine	182.0677	304.5	[M+H]+	10010182.2	9491848.95	↓
163	Propylthiouracil	153.0419	319.8	[M+H-H2O]+	45391486.1	62261495.7	↑
164	N-Acetylhistidine	198.0871	76.1	[M+H]+	162337460	136211085	↓
165	N6-Acetyl-L-lysine	189.1236	124.2	[M+H]+	51340937.3	51202591.5	↓
166	Caryophyllene alpha-oxide	203.1798	827.3	[M+H-H2O]+	3907051.22	4459476.93	↑
167	Myristoleic acid	209.1904	814.5	[M+H-H2O]+	3805269.01	5830701.45	↑
168	Bergapten	216.0425	952.8	[M]+	4694430.46	3765583.85	↓
169	gamma-Glutamylalanine	219.0952	774.9	[M+H]+	4371375.02	1044602.26	↓
170	Ergothioneine	230.0996	103.6	[M]+	51113329	55094205.9	↑
171	Tolmetin	258.1158	490.7	[M+H]+	913648.698	497104.506	↓
172	Daidzein	255.0654	618.1	[M+H]+	1618963.14	1175692.76	↓
173	Stearidonic acid	277.2163	812	[M+H]+	21672028.5	21533782.2	↓
174	Oleamide	282.2794	917.2	[M+H]+	272870337	204310880	↓
175	Octadecanamide	284.2946	927.4	[M+H]+	95690855.3	123924549	↑
176	Inosine	269.0881	232.7	[M+H]+	378068001	279122906	↓
177	Aspartame	295.1291	424	[M+H]+	13351889.6	9113425.07	↓
178	Sulfamethazine	279.0934	754.1	[M+H]+	4813576.21	3007481.24	↓
179	Exemestane	279.1703	487.9	[M+H-H2O]+	8811234.91	8000869.47	↓
180	Norethindrone	281.1904	714.3	[M+H-H2O]+	1406981.91	1430655.41	↑
181	Etiocholanolone	291.2325	857.9	[M+H]+	15448074.2	19553861.6	↑
182	N-Acetyl-a-neuraminic acid	310.1134	99.1	[M+H]+	20850983.9	19467884.9	↓
183	9-OxoODE	295.2261	699.2	[M+H]+	21155382.1	24653047.6	↑
184	Oxycodone	298.1459	551.7	[M+H-H2O]+	4532298.7	2811964.08	↓
185	Metoclopramide	300.1447	609.5	[M+H]+	5315984.84	5496166.32	↑
186	9-cis-Retinoic acid	301.2165	784	[M+H]+	6934193.16	5924382.34	↓
187	Palmitoylethanolamide	300.29	954.8	[M+H]+	328629844	114710615	↓
188	Sphingosine	300.2901	776.9	[M+H]+	13558247.8	13482077	↓
189	Fluvoxamine	319.167	449.5	[M+H]+	1421779.71	1781442.4	↑
190	8-HETE	303.2319	876.1	[M+H-H2O]+	17800215.5	72442655.8	↑
191	12-KETE	318.228	570.4	[M]+	1998359.23	2885420.72	↑
192	Prostaglandin B1	319.2262	713.1	[M+H-H2O]+	190151.769	3216233.85	↑
193	20-HETE	320.2307	732.2	[M]+	1454138.15	894234.476	↓
194	UMP	325.0489	112.9	[M+H]+	31620986	35904025.5	↑
195	Ethyl icosapentate	331.2631	846.6	[M+H]+	11778908	44914920.9	↑
196	Anandamide	348.2891	912.7	[M+H]+	46232158.8	53157952.4	↑
197	Prostaglandin H2	335.2218	714.1	[M+H-H2O]+	21757829.2	16890931.3	↓
198	Prostaglandin B2	335.2199	813.3	[M+H]+	105118032	65418263.9	↓
199	Prostaglandin D2	335.2218	788.7	[M+H-H2O]+	15756345.6	9564918.19	↓
200	13,14-Dihydro-15-keto-PGE2	335.2217	725.3	[M+H-H2O]+	9398139.39	10204112	↑
201	Prostaglandin F3a	335.2218	686.8	[M+H-H2O]+	23864085.2	12321354.3	↓
202	Prostaglandin E1	337.2375	729.5	[M+H-H2O]+	2974211.96	3044520.04	↑
203	AMP	348.0695	121.7	[M+H]+	401122111	800444016	↑
204	3'-AMP	348.0695	100.7	[M+H]+	53740194.8	46688979.2	↓

205	Lathosterol	369.3523	957.9	[M+H-H2O]+	34702978.2	32722148.5	↓
206	Gamma-Tocotrienol	410.3184	929	[M]+	17665120.6	13195327.7	↓
207	beta-Sitosterol	397.3826	965.4	[M+H-H2O]+	17085354.8	23152323.9	↑
208	1-palmitoylglycerophosphocholine	496.3405	791.7	[M]+	158341784	134243087	↓
209	NAD	664.1226	124.4	[M]+	10210923.9	8193059.86	↓
210	allopurinol	137.0447	386.5	[M+H]+	93590982	107522376	↑
211	Fludrocortisone	380.2085	929.3	[M]+	18864164.6	22842103.4	↑
212	Cafestol	317.2118	788.9	[M+H]+	9249521.03	3752416	↓
213	O-Acetylcarnitine	205.1269	124.4	[M+H]+	341979373	303439470	↓
214	N-Acetyl-L-phenylalanine	207.0954	702.3	[M]+	19630761.8	27445524.3	↑
215	Dethiobiotin	215.1397	249.3	[M+H]+	14151643.5	15263419.3	↑
216	(-)-Epigallocatechin	306.0761	166.8	[M]+	15050866.9	14833663.6	↓
217	Di-2-propenyl sulfide	115.0545	399.1	[M+H]+	67061814.3	69570699.2	↑
218	6-Hydroxyhexanoic acid	133.0845	399.1	[M+H]+	10741688.3	9181395.42	↓
219	2-Phenylacetamide	136.0762	429	[M+H]+	2129181.04	4056476.21	↑
220	Indolepyruvate	204.0696	439.6	[M+H]+	1012680.82	701142.528	↓
221	Propionylcarnitine	218.1389	269.7	[M+H]+	1085916626	1212896999	↑
222	Gamma-Linolenic acid	279.2313	841.4	[M+H]+	15497411.9	10870231.6	↓
223	L-Octanoylcarnitine	288.2173	621.1	[M+H]+	251439681	94566079.2	↓
224	Liquiritin	401.1304	282.8	[M+H-H2O]+	19389215.5	13916691.1	↓
225	Myriocin	384.2742	708.5	[M+H-H2O]+	5330114.03	4633194.38	↓
226	Adenylsuccinic acid	464.0804	312.7	[M+H]+	9766154.45	4853421.12	↓
227	N-Methyl-2-pyrrolidinone	100.0762	340.3	[M+H]+	6751931.52	3749102.26	↓
228	4-Hydroxybenzaldehyde	123.0444	126.5	[M+H]+	80418716.9	61806255.3	↓
229	8-Hydroxyquinoline	146.0595	399.1	[M+H]+	540858233	452701608	↓
230	Isoquinoline	130.0659	399.3	[M+H]+	104519964	73909409.3	↓
231	2-Naphthylamine	144.0813	441.4	[M+H]+	23233278.4	16862728.6	↓
232	1,5-Naphthalenediamine	159.092	399.1	[M+H]+	99991302.3	116564214	↑
233	3,4-Dihydroxyphenylpropanoate	165.0547	126.2	[M+H-H2O]+	213889475	222661669	↑
234	Jasmone	165.1278	497.9	[M+H]+	1487620.99	1952954.79	↑
235	Glycyl-leucine	189.1244	366	[M+H]+	58544907.4	37932156	↓
236	Nootkatone	219.1746	868.3	[M+H]+	10602495.6	13991682.6	↑
237	N-Acetyl-D-galactosamine	204.0869	99.3	[M+H-H2O]+	25922877	9809069.37	↓
238	Diethylpropion	206.1525	117.7	[M+H]+	22033660.7	27430270.4	↑
239	3,3'-Dimethoxybenzidine	244.1195	974.6	[M]+	13707511	22494644.3	↑
240	(-)-Columbianetin	247.0935	741.3	[M+H]+	2081500.49	2428677.39	↑
241	Penbutolol	292.2273	629.2	[M+H]+	1317107.65	1510618.81	↑
242	N6-(L-1,3-Dicarboxypropyl)-L-lysine	277.1396	86.8	[M+H]+	24351429.2	17563490.4	↓
243	(9Z,11E,13E)-Octadecatrienoic acid	279.2316	706.3	[M+H]+	13061035.8	4620072.81	↓
244	N7-Methylguanosine	298.115	323.6	[M]+	2964272.76	2776330.4	↓
245	Tridemorph	298.311	944.7	[M+H]+	37073605.8	49624192.5	↑
246	Diosmetin	301.064	83	[M+H]+	73408915.3	49810407	↓
247	Oleoylethanolamide	326.3059	970.4	[M+H]+	89763839.5	98894302.9	↑
248	Testosterone cypionate	412.3043	742.4	[M]+	8577244.8	14448133.7	↑

249	Raffinose	487.1651	99.3	[M+H-H2O]+	39894212.6	45273862.4	↑
250	2-Hydroxybutyric acid	104.0534	125.3	[M]+	10873425.1	11120313.7	↑
251	2-Phenylethanol	105.0703	805.6	[M+H-H2O]+	5413083.97	6463208.58	↑
252	para-Phenylenediamine	109.0766	84.8	[M+H]+	168980009	198447449	↑
253	5-Methyl-2-furancarboxaldehyde	110.0353	149.3	[M]+	201738790	182825792	↓
254	Imidazole-4-acetaldehyde	111.0558	45.8	[M+H]+	43296964.9	26695431.6	↓
255	Monomethyl sulfate	111.9847	62.3	[M]+	5918541.39	6576459.93	↑
256	1-Pyrroline-5-carboxylic acid	113.0462	61.9	[M]+	2594464.04	2379452.32	↓
257	Agmatine	113.1158	67.5	[M+H-H2O]+	5801321.4	5494225.72	↓
258	1-Pyrroline-2-carboxylic acid	114.0662	449.5	[M+H]+	70240037.8	90990345.7	↑
259	Epsilon-caprolactam	114.0918	423.6	[M+H]+	10203163.9	5025439.53	↓
260	L-3-Cyanoalanine	115.0507	94.9	[M+H]+	24030858.2	16152354.4	↓
261	1-Deoxy-D-xylulose	117.0571	188.7	[M+H-H2O]+	13178954.7	12600738	↓
262	5-Hydroxypentanoic acid	118.0655	162.7	[M]+	18589164.6	49839574.5	↑
263	2-Methylserine	119.0501	125.9	[M]+	83523339.4	79355789.2	↓
264	Styrene Oxide	120.0452	341.4	[M]+	4718971.21	2919478.46	↓
265	5-Hydroxypyrazinamide	124.0478	188.1	[M+H]+	5649242.24	4332131.13	↓
266	(R)-5,6-Dihydrothymine	128.0716	62.3	[M]+	1030461.24	832520.721	↓
267	5-Amino-2-oxopentanoic acid	131.0537	157.6	[M]+	12660297.7	9444487.77	↓
268	D-allo-Isoleucine	132.1021	365.8	[M+H]+	11774183.3	17635429.5	↑
269	4-Hydroxyphenylacetaldehyde	136.0618	675	[M]+	1986400.13	2694027.6	↑
270	Propanoyl phosphate	153.9936	979.1	[M]+	42437130.7	33609729.4	↓
271	Trigonelline	137.045	963.3	[M]+	29423392	37568417.2	↑
272	4-Hydroxybenzoic acid	138.0297	284	[M]+	158263778	74447135.5	↓
273	3-Hydroxybenzoic acid	138.0299	192.7	[M]+	5424015.73	5104823.27	↓
274	(3S,5S)-Carbapenam-3-carboxylic acid	155.0566	283.9	[M]+	10415961.2	17117484.1	↑
275	Acetylphosphate	139.9817	294.2	[M]+	57346579.5	60905434.7	↑
276	5-(2-Hydroxyethyl)-4-methylthiazole	143.0399	987.6	[M]+	18193577	15523908.3	↓
277	Ectoine	143.0818	100.6	[M+H]+	30203497.2	25432196.7	↓
278	2-Keto-glutaramic acid	145.0499	89.6	[M]+	27312354.3	43791730.3	↑
279	Acetylcholine	146.118	393.6	[M]+	9492211.5	8393435.53	↓
280	Coumarin	147.0444	126.3	[M+H]+	77037509.3	66646381.6	↓
281	2-Dehydropantoate	147.0637	399	[M+H]+	74361306.2	47162426.9	↓
282	(S)-2-Methylmalate	148.0429	115.8	[M]+	82540180	79898065.3	↓
283	2-Oxo-4-methylthiobutanoic acid	149.0234	722	[M+H]+	8579366.41	14590301.9	↑
284	Acetaminophen	151.0618	330.6	[M]+	31238369.4	51118543.7	↑
285	p-Hydroxyphenylacetic acid	153.0406	228.5	[M+H]+	32966177	27757233.6	↓
286	2-Biphenylol	153.066	84.7	[M+H-H2O]+	149488831	162319038	↑
287	2-Pyrocatechuic acid	154.0415	401.2	[M]+	1474129388	1555113254	↑
288	Imidazol-5-yl-pyruvate	155.0455	401.2	[M+H]+	207016086	207428367	↑
289	(1R,6S)-6-Amino-5-oxocyclohex-2-ene-1-carboxylate	155.0566	232.2	[M]+	3099161.12	3756028.95	↑
290	2,6-Dimethoxyphenol	155.0706	516.1	[M+H]+	2637885.47	2978454.21	↑
291	Scopoline	156.1023	108.2	[M+H]+	13398171.1	12761092.4	↓
292	5-Acetamidovalerate	159.0839	539.8	[M]+	445143.083	755127.547	↑

293	S-Methyl-L-methionine	164.074	330.6	[M]+	2472717.25	2471748.43	↓
294	7-Methylguanine	166.0727	323.6	[M+H]+	25125132.8	21655345.9	↓
295	Tropate	167.0567	332.2	[M+H]+	1951846.81	1911483.61	↓
296	D-synephrine	168.0914	340.5	[M+H]+	8229882.35	9941763.16	↑
297	(S)-4-Hydroxymandelate	169.046	72	[M+H]+	53565906.5	44470968.2	↓
298	(R)-2-O-Sulfolactate	169.977	618.7	[M]+	200314101	187614405	↓
299	3-Indoleacrylate	170.0604	399	[M+H-H2O]+	134446143	228115387	↑
300	Diphenylamine	170.0976	890.3	[M+H]+	21093790.1	15537918.6	↓
301	N-Butyryl-L-homoserine lactone	172.0972	439.9	[M+H]+	4554103.76	3020463.03	↓
302	2-Oxoarginine	173.0814	378.2	[M]+	30367534.7	17177014.7	↓
303	Norselegiline	174.1281	904.3	[M+H]+	4840441.22	6561137.98	↑
304	N-Acetyl-L-glutamate 5-semialdehyde	174.0878	106.6	[M+H]+	378819.367	16848490.1	↑
305	N-Formyl-L-glutamic acid	176.0744	371.9	[M+H]+	1374660.75	1526822.15	↑
306	Citrulline	176.1038	111.3	[M+H]+	23544065.7	19363014.2	↓
307	Methyleugenol	179.107	799.6	[M+H]+	10791316	16450355.4	↑
308	L-Homophenylalanine	180.1024	425.6	[M+H]+	2826149.67	3274314.01	↑
309	(S)-beta-Tyrosine	182.0815	316.7	[M+H]+	11259848.6	7432227.26	↓
310	Se-Methylselenocysteine	182.9847	60.6	[M]+	19922787.3	21791036.1	↑
311	Hydroxyphenyllactic acid	183.0843	189.3	[M+H]+	50226135	49242894.2	↓
312	Phosphorylcholine	184.0734	86.8	[M+H]+	1698741118	1463978006	↓
313	5-Guanidino-3-methyl-2-oxopentanoate	187.1085	356.1	[M]+	2664956.15	5193175.04	↑
314	Diaminopimelic acid	190.1078	444.4	[M]+	546054.323	904332.356	↑
315	N,N-Diethyl-m-toluamide	192.1386	725.3	[M+H]+	1620486.55	2259847.97	↑
316	Sequoyitol	194.0816	304.1	[M]+	10795065.2	13228805.3	↑
317	(-)-Salsoline	194.118	973.4	[M+H]+	16561770	28334657.8	↑
318	3,4-Methylenedioxyamphetamine	194.1183	311.7	[M+H]+	33908745.7	38009034.1	↑
319	3-Methyl-L-tyrosine	195.0882	441.3	[M]+	2658803.04	4438785.59	↑
320	gamma-Glutamyl-beta-aminopropiononitrile	200.0923	314.7	[M+H]+	2938031.91	3038441.55	↑
321	Asymmetric dimethylarginine	203.1506	94.9	[M+H]+	89720962.6	82161628.6	↓
322	N5-(L-1-Carboxyethyl)-L-ornithine	204.1235	514.4	[M]+	9446216.03	5304754.14	↓
323	3,4-Dihydro-2-methylene-3-oxo-2H-1,4-benzoxazine-5-carboxylate	206.0487	168.4	[M+H]+	6928975.12	7323952.65	↑
324	Indolelactic acid	206.0817	550.1	[M+H]+	3601497.62	3887762.04	↑
325	N-a-Acetylcitrulline	218.1135	89.5	[M+H]+	101220352	102518569	↑
326	Capsidiol	219.1746	676.3	[M+H-H2O]+	61878903.1	58485997.1	↓
327	Pyrimidodiazepine	221.0925	399	[M]+	5990204.43	2670235.13	↓
328	Prephenate	226.0683	842.2	[M]+	25615284.6	24132356.5	↓
329	beta-Alanyl-L-arginine	245.15	320.6	[M]+	5353479.46	5881618.08	↑
330	Confertifolin	234.1623	818.2	[M]+	1589644.93	2513966.42	↑
331	4a-Carbinolamine tetrahydrobiopterin	239.1031	275.4	[M]+	484646.45	415536.83	↓
332	Linatine	260.1239	103.5	[M+H]+	12654132.2	15287524.6	↑
333	1-(3,4-Dihydroxyphenyl)-5-hydroxy-3-decanone	281.1496	359.5	[M+H]+	7742718.87	6246501.38	↓
334	Retinol	269.2265	826.6	[M+H-H2O]+	5957287.69	5879556.06	↓
335	Medicarpin	270.0905	174.6	[M]+	10126289.6	12594724.2	↑
336	(2R,3R)-3-Methylornithinyl-N6-lysine	275.2084	67.9	[M+H]+	682246.743	6789654.92	↑

337	4-Hydroxycinnamoylagmatine	276.1447	237.4	[M]+	8310000.63	25374140.3	↑
338	Cyclopeptine	281.1138	332.3	[M+H]+	8144222.48	6590309.24	↓
339	3-Ketosphingosine	280.2638	847.4	[M+H-H2O]+	18987735.6	20058188.9	↑
340	Sphinganine	284.2943	781	[M+H-H2O]+	12004774.4	12458147.6	↑
341	Cyclopentolate	292.1908	601.8	[M+H]+	917293.733	561774.378	↓
342	12-OPDA	292.2015	582.5	[M]+	4052639.8	4974328.66	↑
343	9(S)-HPOT	293.2115	835.1	[M+H-H2O]+	8531193.82	2858323.77	↓
344	13-OxoODE	295.2276	869.1	[M+H]+	5962101.18	9806082.65	↑
345	3-Dehydrosphinganine	300.2901	816.8	[M+H]+	6594803.16	3475422.11	↓
346	N-Acetylaspartylglutamic acid	305.0985	196.1	[M+H]+	7949515.66	3352608.98	↓
347	Methoprene	311.2584	807.4	[M+H]+	8921680.57	8185578.27	↓
348	Progesterone	314.2325	674	[M]+	1562409.68	1125958.11	↓
349	Decanoyl-L-carnitine	316.2481	704.7	[M+H]+	76631836	43195915.4	↓
350	8,9-DiHETrE	321.2426	863	[M+H-H2O]+	6769857.71	6455579.88	↓
351	gamma-L-Glutamyl-L-cysteinyl-beta-alanine	322.0772	194.2	[M+H]+	8691804.98	10331724.2	↑
352	Prostaglandin E2	335.2218	668.2	[M+H-H2O]+	7254837.47	2036200.68	↓
353	2-Hydroxy-6-pentadecylbenzoic acid	349.2735	902.8	[M+H]+	12393684.6	6624485.6	↓
354	Alpha-Linolenoyl ethanolamide	350.3059	868.9	[M+H]+	73339086.9	126889840	↑
355	(13E)-11a-Hydroxy-9,15-dioxoprost-13-enoic acid	353.2309	749.1	[M+H]+	13025954.8	5842124.2	↓
356	11b-PGF2a	354.2639	699.5	[M]+	1071537.82	559852.528	↓
357	Nitrendipine	361.1392	554	[M+H]+	5599444.61	3805481.51	↓
358	1-Arachidonoylglycerol	361.2736	826.8	[M+H-H2O]+	393258684	400743144	↑
359	Sphinganine 1-phosphate	364.2505	847.6	[M+H-H2O]+	13984188.4	9582428.83	↓
360	Docosatetraenoyl Ethanolamide	376.3104	802.7	[M+H]+	40743373.5	75759921.2	↑
361	Dihydroanhydropodorhizol	383.1503	228.7	[M+H-H2O]+	22538413.5	11232844.8	↓
362	3-Geranylgeranylindole	390.3084	874.5	[M+H]+	12585886.5	7895994.09	↓
363	Yamogenin	397.2945	918	[M+H-H2O]+	53510735.5	34588185.1	↓
364	14alpha-Hydroxy-5beta-cholest-7-ene-3,6-dione	414.3005	709.6	[M]+	2667211.56	3019906.47	↑
365	3-Keto-4-methylzymosterol	397.3827	929.4	[M+H]+	7924647.87	10093140.6	↑
366	Palmitoyl-L-carnitine	400.3422	810.4	[M+H]+	664139618	1419134210	↑
367	Delta-Tocopherol	403.3501	810.4	[M+H]+	1951639.86	3541368.42	↑
368	Avenasterol	412.342	937.7	[M]+	3560786.21	5381157.52	↑
369	Paspalicine	418.2432	827.5	[M+H]+	2951432.39	2272028.95	↓
370	HC-toxin	437.2412	463.7	[M+H]+	705017.557	570347.575	↓
371	4a-Carboxy-4b-methyl-5a-cholesta-8,24-dien-3b-ol	442.3535	918	[M]+	33038827	32426766.1	↓
372	Retinoyl b-glucuronide	476.2816	804.2	[M]+	10369031.6	9673145.66	↓
373	3-Dehydroecdysone	462.2729	712.3	[M]+	3972441.99	653153.074	↓
374	Antibiotic JI-20A	482.3231	779.5	[M+H]+	37815309.7	62846257.2	↑
375	3-Epiecdysone	464.3072	721.6	[M]+	40231165	27247692.3	↓
376	Cytochalasin B	480.2776	674.4	[M+H]+	7237588.07	8515540.78	↑
377	Citicoline	489.1092	90.8	[M+H]+	213915319	157696336	↓
378	Mibefradil	495.2981	903.9	[M]+	195404731	87684359.9	↓
379	Cucurbitacin E	556.3011	876.3	[M]+	4335330.99	7857855.64	↑
380	5-Oxoavermectin "1b" aglycone	568.3391	830.4	[M]+	218913760	76267871	↓

381	Avermectin B1b aglycone	570.3581	872	[M]+	332323906	279905126	↓

Table S3 Identified for 191 metabolomics in negative ionization mode.

No.	Name	m/z	Rt(s)	Addut	CON	PM	Variation
1	D-Proline	114.0549	127.5	[M-H]-	8065104.92	7061485.17	↓
2	beta-D-3-Ribofuranosyluric acid	299.0628	128.1	[M-H]-	5654349.99	2865122.45	↓
3	UDP	384.9936	94	[M-H2O-H]-	1561897494	2016207909	↑
4	3-Dehydro-4-phospho-D-erythronate	212.9801	72.9	[M-H]-	7743442.07	3794135.94	↓
5	Sedoheptulose 7-phosphate	290.0364	76.3	[M]-	58920883	66744810.6	↑
6	Sedoheptulose 1,7-bisphosphate	368.9983	67.5	[M-H]-	17501221.1	24350258.8	↑
7	6-Acetyl-D-glucose	221.0664	96.5	[M-H]-	125931784	78119374.3	↓
8	beta-1,2-Mannobiose	341.1008	422.1	[M-H]-	898119.337	420181.551	↓
9	S-Glutathionyl-L-cysteine	425.0789	81.1	[M-H]-	267690830	269927099	↑
10	Oxidized glutathione	611.1375	80	[M-H]-	767322747	691449477	↓
11	3'-Ketolactose	339.0944	345.9	[M-H]-	464171.217	1149116.89	↑
12	Biotinyl-5'-AMP	573.1429	97.4	[M]-	1629075.59	1659438.48	↑
13	2-O-[2-O-(alpha-D-Mannopyranosyl)-alpha-D-glucopyranosyl]-3-phospho-D-glycerate	509.0918	95.5	[M-H]-	31429530.1	19270690.3	↓
14	Phosphoadenosine phosphosulfate	505.9855	86.7	[M-H]-	33686621.2	30097705.6	↓
15	N-Acetyl-D-Glucosamine 6-Phosphate	300.0497	78.3	[M-H]-	92897006.5	93057378.3	↑
16	9,12,13-TriHOME	329.2334	642.8	[M-H]-	28733139.2	8922370.53	↓
17	9,10-Dihydroxy-12,13-epoxyoctadecanoate	329.2339	624	[M-H]-	35688930.2	19374791.9	↓
18	Dimethylglycine	102.0548	80.8	[M-H]-	18971836.6	14019118.5	↓
19	L-Serine	104.0341	86.1	[M-H]-	60538618	40180694.1	↓
20	Glyceric acid	105.0181	82.5	[M-H]-	142579812	137270754	↓
21	L-Aspartic acid	132.0291	80.7	[M-H]-	158796987	274421574	↓
22	Caproic acid	114.9332	922.6	[M-H]-	1427361004	1982528372	↑
23	Fumaric acid	115.0025	74.4	[M-H]-	252592181	276268514	↑
24	5-Aminopentanoic acid	116.9273	141	[M]-	47980363.4	75701688.7	↑
25	trans-1,2-Cyclohexanediol	115.9194	931.1	[M]-	56713781.8	320238240	↑
26	Guanidinoacetate	116.0494	366.1	[M-H]-	9983227.81	30975784.5	↑
27	L-Valine	116.0702	112.8	[M-H]-	39457707.4	73944910.3	↑
28	3-Methylthiopropionic acid	119.049	155.1	[M-H]-	3631528.63	3732274.69	↑
29	Erythritol	121.0283	498.5	[M-H]-	23508713.3	22369414.4	↓
30	Taurine	124.0063	86.6	[M-H]-	927163510	692215126	↓
31	Pyroglutamic acid	128.0339	90.5	[M-H]-	621074424	1162878357	↑
32	Creatine	130.0611	96.7	[M-H]-	823602263	709836639	↓
33	Leucine	130.0862	131.2	[M-H]-	174741183	165421503	↓
34	L-Malic acid	133.0128	74.3	[M-H]-	2964417295	2100516378	↓
35	Adenine	134.0457	921.7	[M-H]-	37150230.8	25881936.4	↓
36	Phenyl acetate	134.8944	823.9	[M-H]-	70769877.7	47314744.3	↓
37	Hypoxanthine	135.0292	130.5	[M-H]-	1186986534	851826093	↓
38	O-Phosphoethanolamine	140.0107	80.9	[M-H]-	82265834.3	63090752.2	↓

39	Aminoadipic acid	160.0606	81	[M-H]-	35262714.4	10538041.3	↓
40	Spermidine	143.9145	51.2	[M-H]-	10677711.3	9064416.05	↓
41	Dimethylbenzimidazole	146.0813	156.4	[M]-	3918171.82	2540415.04	↓
42	2',4'-Dihydroxyacetophenone	151.0391	609	[M-H]-	1741177.85	1238099.71	↓
43	D-Xylitol	151.0603	91.8	[M-H]-	21149909.5	13729081.8	↓
44	Pelargonic acid	157.123	852.1	[M-H]-	270640691	373802825	↑
45	Oxoadipic acid	158.9775	121.9	[M-H]-	98649665	69800578.3	↓
46	Nicotine	160.9347	862.5	[M-H]-	6834636.9	12662421.3	↑
47	D-Galactose	161.0447	96.1	[M-H2O-H]-	244901192	797643971	↑
48	Phenylpyruvic acid	163.0402	394.2	[M-H]-	3808826.84	2479455.72	↓
49	D-Phenylalanine	165.0184	985.7	[M]-	25268452.8	17649947.3	↓
50	Phthalic acid	165.0407	884.8	[M-H]-	165704190	95237953.7	↓
51	Uric acid	167.0201	99.4	[M-H]-	527030078	529610552	↑
52	N-Acetylleucine	172.0967	337.2	[M-H]-	2727687.78	1545630.09	↓
53	Shikimic acid	172.9908	386.8	[M-H]-	6175320.23	2474031.75	↓
54	N-Formyl-L-methionine	176.0378	132.2	[M-H]-	11985013.9	10574198.6	↓
55	Gluconic acid	195.0503	82.4	[M-H]-	199489257	212757195	↑
56	Paraxanthine	179.9882	950.2	[M]-	64596168.6	65905304.4	↑
57	D-Mannose	179.9884	751.9	[M]-	18646847.9	13873724.7	↓
58	Azelaic acid	187.0958	986.4	[M-H]-	48418965.6	36663194.3	↓
59	Homo-L-arginine	188.1116	126.4	[M]-	3111995.05	2334969.54	↓
60	Citric acid	191.0192	69.5	[M-H]-	420799062	213484985	↓
61	L-Dopa	196.061	154.2	[M-H]-	751046.422	707135.636	↓
62	N-Acetylserotonin	199.1699	880.8	[M-H2O-H]-	77981204.4	33255760.2	↓
63	L-Cystathionine	221.0598	83	[M-H]-	62796448.5	45464470.2	↓
64	Methyl jasmonate	205.159	888.1	[M-H2O-H]-	14199472.6	15111521.8	↑
65	Carnosine	225.0973	98.1	[M-H]-	1.1132E+10	1.8481E+10	↑
66	Methyldopa	211.1337	631.3	[M]-	5169635.78	3400799.12	↓
67	Pantothenic acid	218.1023	129.5	[M-H]-	1274619242	950943452	↓
68	N-Acetylmannosamine	220.0824	100.9	[M-H]-	10619255.7	7925143.34	↓
69	N-Acetyl-D-glucosamine	221.1544	675	[M]-	8323301.33	5769952.56	↓
70	Thymidine	223.028	855.8	[M-H2O-H]-	40576961.3	30153386.7	↓
71	Myristic acid	227.2015	953.1	[M-H]-	181997949	168260912	↓
72	gamma-Glutamylcysteine	248.9604	89.6	[M-H]-	6112916.86	7169348.44	↑
73	Equol	241.1811	762	[M-H]-	25341457.8	16532274.7	↓
74	Uridine	243.0619	130.5	[M-H]-	110678942	109813095	↓
75	Pyridoxal phosphate	247.1702	868.6	[M]-	6829159.23	8787475.28	↑
76	Deoxyguanosine	266.0898	298.3	[M-H]-	1381843.07	857884.93	↓
77	2'-Deoxyadenosine	251.0958	624.1	[M]-	11122142.4	7923799.79	↓
78	5,7-Dihydroxyflavone	253.0507	614.6	[M-H]-	999802.401	1118291.41	↑
79	Stearic acid	284.2645	931.2	[M]-	868215874	876226354	↑
80	Formononetin	267.1918	265.4	[M-H]-	5928860.39	5446139.72	↓
81	Dehydroepiandrosterone	269.2125	845.6	[M-H2O-H]-	265690147	196180864	↓
82	17a-Estradiol	271.2279	826.3	[M-H]-	77329635	66113896.7	↓

83	Epiandrosterone	271.2279	890.6	[M-H2O-H]-	669889498	143524916	↓
84	6-Phosphogluconic acid	257.0069	75.7	[M-H2O-H]-	26379588.2	16339943.8	↓
85	Kaempferide	299.2547	755.2	[M-H]-	44149872.6	38412406.7	↓
86	2-Methoxyestradiol	283.1696	888.9	[M-H2O-H]-	150081945	87213745.4	↓
87	Ribose 1,5-bisphosphate	309.1733	786.8	[M-H]-	71830022	59936894.7	↓
88	Nonadecanoic acid	297.2411	743.6	[M-H]-	14837973.6	14555143.9	↓
89	all-trans-Retinoic acid	300.205	906	[M]-	1153286.36	5859874.11	↑
90	Arachidic acid	311.2957	942.3	[M-H]-	13457217.8	7430420.31	↓
91	dGMP	346.0552	119.2	[M-H]-	178562515	182743348	↑
92	Fructose 1,6-bisphosphate	338.989	67.6	[M-H]-	146653469	140693984	↓
93	Cellobiose	341.1093	96.7	[M-H]-	385394834	283309497	↓
94	Deoxycorticosterone acetate	371.0656	813.6	[M-H]-	2298047.79	2670252.66	↑
95	Xanthylic acid	363.0339	74.2	[M-H]-	34019856.6	14629470.8	↓
96	S-Adenosylhomocysteine	383.1138	308.3	[M-H]-	32853730.9	31931827.9	↓
97	Chenodeoxycholic acid	391.285	791	[M-H]-	9925957.1	8074022.64	↓
98	ADP	426.0202	94.3	[M-H]-	113995968	176237956	↑
99	Puerarin	415.1038	430.6	[M-H]-	5259137.25	3258789.46	↓
100	Folic acid	441.2513	808.9	[M]-	21080827.5	11336494.1	↓
101	Glycochenodeoxycholic acid	448.3067	653.1	[M-H]-	979818.806	1773857.91	↑
102	Glycocholic acid	464.3018	602.9	[M-H]-	1966528.3	1355149.57	↓
103	(R)-3-Hydroxybutyric acid	103.0389	93.5	[M-H]-	90406135.4	84647925.7	↓
104	Adipic acid	127.0388	126.2	[M-H2O-H]-	27001339.1	41734345.2	↑
105	3-Methyl-2-oxovaleric acid	129.0547	331.6	[M-H]-	26637168.8	18671900.7	↓
106	cis-4-Hydroxy-D-proline	130.0499	89.9	[M-H]-	92861285.8	64290331.5	↓
107	Ribitol	133.0496	98	[M-H2O-H]-	20901964.6	14707730.2	↓
108	4-Nitrophenol	138.0192	608.9	[M-H]-	6887004.27	10766793.4	↑
109	Pimelic acid	141.0547	81.8	[M-H2O-H]-	21703645.1	12218886.5	↓
110	Aminosalicylic Acid	152.0352	701	[M-H]-	2753615.89	1482005.04	↓
111	Pyrophosphate	176.9351	78.5	[M-H]-	3107638190	2533621006	↓
112	m-Coumaric acid	163.0391	154.8	[M-H]-	1928568.83	2164029.3	↑
113	Formylanthranilic acid	164.0348	349.3	[M-H]-	728280.543	780018.248	↑
114	4-Pyridoxic acid	182.0445	338.4	[M-H]-	95739742.9	94399100.8	↓
115	1-Methylxanthine	165.0397	967.1	[M-H]-	52389671.7	26930704.9	↓
116	Phosphoenolpyruvic acid	166.9743	70.3	[M-H]-	6758193.59	6892673.64	↑
117	Dihydroxyacetone phosphate	168.9899	75.3	[M-H]-	59704739.4	60219268.6	↑
118	N-Acetylornithine	173.0982	125.9	[M-H]-	20751860.1	16272839.1	↓
119	D-Erythrose 4-phosphate	199.0008	75.9	[M-H]-	280079114	214838997	↓
120	L-Homocysteic acid	182.0122	128.9	[M-H]-	78562901.4	9261985.85	↓
121	(2R)-2-Hydroxy-3-(phosphonatooxy)propanoate	184.9849	71.7	[M-H]-	133996141	100468802	↓
122	Pantothenol	204.1238	351.7	[M-H]-	1073549.6	773212.071	↓
123	Xylulose 5-phosphate	211.0008	78.3	[M-H2O-H]-	76039709.4	51029109.7	↓
124	Dodecanedioic acid	211.1324	805.8	[M-H2O-H]-	14263619.4	14641504.2	↑
125	D-Arabinose 5-phosphate	229.0114	77.1	[M-H]-	437327103	253334823	↓

126	Gemfibrozil	249.1496	736.6	[M-H]-	3064224.27	1166750.15	↓
127	Glucose 6-phosphate	260.0252	75.8	[M]-	204498129	285712691	↑
128	Fructose 1-phosphate	241.0117	77.9	[M-H2O-H]-	72691235.4	29785253.7	↓
129	Deoxyinosine	251.0787	338.4	[M-H]-	1031040.47	975943.941	↓
130	16-Hydroxy hexadecanoic acid	271.2281	922.4	[M-H]-	57766384.3	44854066.3	↓
131	[8]-Shogaol	275.169	768.1	[M-H]-	37366640.1	7749922.47	↓
132	Practolol	265.1577	709.5	[M-H]-	3358648.78	254735.041	↓
133	9,10-Epoxyoctadecenoic acid	295.228	839.5	[M-H]-	3556278141	3091278585	↓
134	10E,12Z-Octadecadienoic acid	280.2365	780.6	[M]-	3286889308	1440989326	↓
135	Gingerol	293.1766	713.9	[M-H]-	5188620.25	1509314.55	↓
136	13-L-Hydroperoxylinoleic acid	293.2124	855.1	[M-H2O-H]-	231494962	231334506	↓
137	13S-hydroxyoctadecadienoic acid	295.2307	714	[M-H]-	4833713.1	6445002.79	↑
138	16(R)-HETE	319.2281	844.5	[M-H]-	417014483	405036735	↓
139	19(S)-HETE	301.2169	855.8	[M-H2O-H]-	47949275.6	30446255.9	↓
140	dTMP	321.0482	123.1	[M-H]-	7350666.17	3092243.14	↓
141	6beta-Hydroxytestosterone	303.2	808.5	[M-H]-	29627194.5	8470476.25	↓
142	Arachidonic acid	303.2333	862.4	[M-H]-	120913624	75448081	↓
143	Eicosadienoic acid	308.2675	949.9	[M]-	161112754	156974644	↓
144	11Z-Eicosenoic acid	310.2835	950.3	[M]-	122709057	81782748.7	↓
145	Levonorgestrel	311.2016	892.1	[M-H]-	321676305	211175342	↓
146	9(S)-HPODE	311.2229	779.8	[M-H]-	818312544	359484648	↓
147	9,10-DHOME	313.2382	766.8	[M-H]-	40220401.3	43345700	↑
148	Prostaglandin A2	333.2079	699.4	[M-H]-	2225004.8	1798182.75	↓
149	15-Deoxy-d-12,14-PGJ2	315.197	699.2	[M-H]-	1558632.59	1310964.98	↓
150	5-KETE	317.2119	878	[M-H]-	34319019.4	22072284.5	↓
151	Aesculin	339.0643	958	[M-H]-	11937415	11513628.4	↓
152	Docosapentaenoic acid (22n-3)	329.2483	973.9	[M-H]-	210735171	188666607	↓
153	Adrenic acid	331.2652	932.9	[M-H]-	100111361	75288743.9	↓
154	Prostaglandin A1	335.2233	793.6	[M-H]-	13750209.4	47273575.2	↑
155	14,15-DiHETrE	337.2383	766.9	[M-H]-	4041481.31	3302318.73	↓
156	Prostaglandin E3	349.204	811.2	[M-H]-	5834399.92	3066708.38	↓
157	GMP	362.0496	93.1	[M-H]-	98587333.8	64996515.8	↓
158	Alpha-Tocotrienol	423.3262	882.5	[M-H]-	899434357	456749096	↓
159	FMN	455.0967	385.7	[M-H]-	9374329.12	5411113.14	↓
160	Adenosine diphosphate ribose	540.0536	129	[M-H2O-H]-	30496381	24037712.7	↓
161	Uridine diphosphate glucose	565.0437	80	[M-H]-	12952406.3	19450501.4	↑
162	Uridine diphosphate glucuronic acid	579.0351	70.1	[M-H]-	25375481	16030565.1	↓
163	NADH	664.1227	156.9	[M-H]-	18026005.7	14277136.2	↓
164	FAD	784.1522	356.2	[M-H]-	78055597.8	70207189.8	↓
165	Maltohexaose	989.3319	124.6	[M-H]-	54742573	20418552.2	↓
166	Biochanin A	283.0681	703.4	[M-H]-	10597216.3	7416611.46	↓
167	1H-Indole-3-carboxaldehyde	144.0436	584.4	[M-H]-	31399012.8	21762957.3	↓
168	Citramalic acid	147.029	80.2	[M-H]-	33123906.3	24499207.4	↓
169	4-Hydroxycinnamic acid	163.0392	130.3	[M-H]-	11239831.8	8601980.74	↓

170	2(3H)-Benzothiazolethione	165.9784	671	[M-H]-	1662975.73	921614.599	↓
171	Gallic acid	169.0167	128.1	[M-H]-	3047625.58	2929042.72	↓
172	Beta-Glycerophosphoric acid	171.0056	77.9	[M-H]-	26470567.7	15787660.9	↓
173	Capric acid	171.1383	800.7	[M-H]-	15341791.8	7753310.57	↓
174	2,4-Dinitrophenol	183.0049	459.9	[M-H]-	2805943.07	2516373.64	↓
175	Pentadecanoic acid	241.2175	812.7	[M-H]-	27665715.5	20723714.2	↓
176	Palmitoleic acid	253.2153	727.8	[M-H]-	3613207.17	4748058.52	↑
177	Dibutyl phthalate	277.1449	741.4	[M-H]-	4652289.16	1794823.73	↓
178	Xanthosine	283.0677	149.4	[M-H]-	3156092.68	2342907.25	↓
179	15-KETE	317.212	856.1	[M-H]-	71530332.8	23379192.6	↓
180	dIMP	331.0436	119.4	[M-H]-	10314214.4	6032873.32	↓
181	S-Lactoylglutathione	378.0949	128.6	[M-H]-	11985303.7	8470240.22	↓
182	UDP-D-Xylose	535.0371	80	[M-H]-	11478226.4	5327870.57	↓
183	Phenyllactate	165.0559	344.3	[M-H]-	16903288.3	12686095.2	↓
184	Quinate	173.0447	310.2	[M-H2O-H]-	3436178.56	5384905.32	↑
185	Isopropylparaben	179.0708	740.8	[M-H]-	1073246.23	1242711.7	↑
186	4-Acetylaminobiphenyl	210.0955	484.4	[M-H]-	1007959.91	1058082.88	↑
187	Shikimate 3-phosphate	253.0121	77.8	[M-H]-	3332159.59	2823439.37	↓
188	Hexadecanedioate	285.2072	746.9	[M-H]-	18764940	8463722.15	↓
189	13(S)-HPOT	291.2	770.8	[M-H2O-H]-	1273740.65	1456463.99	↑
190	12,13-DHOME	314.2423	767.3	[M]-	1644170.83	6459802.73	↑
191	EPA (d5)	302.2197	923.4	[M]-	143689596	156691471	↑

Table S4 The details of some flavour compounds of breast muscle in the CON and PM groups

Name	m/z	Rt(s)	Addut	CON	PM	Variation	Odor descriptors
valeric acid	102.0911	533.4	[M]+	3326764.92	2965388.44	↓	Cheesy, acidic
Isovaleric acid	102.0912	377.7	[M]+	5235997.17	4379589.7	↓	Pungent, cheesy, acidic
Heptanoic acid	130.9673	254.6	[M+H]+	12088932.6	7431762.03	↓	Waxy, cheesy, fruity
Phenylacetaldehyde	121.0648	340	[M+H]+	9949676.92	5971588.47	↓	Floral, sweet, fermented
m-Cresol	109.0653	188.6	[M+H]+	8526141.64	8971680.77	↑	Smoky, creamy, medicinal
Phenylacetic acid	136.0495	80.6	[M]+	9337718.64	6480346.53	↓	Sweet, floral
2,3-Butanediol	154.0444	319.5	[M]+	8031221.35	4376043.89	↓	Fruity, creamy, oily
Benzaldehyde	107.0495	746.2	[M+H]+	89130279.9	70907821.4	↓	Almond

Table S5 The details of the significantly enriched KEGG pathway are involved by different metabolites (In the red background).

Tryptophan metabolism	Name	*P*-value	Variation
C00078	L-Tryptophan	0.02259	↓
C00322	2-Oxoadipate	0.0440	↓
C00978	N-Acetylserotonin	0.1008	↓
C00331	Indolepyruvate	0.4443	↓
C05653	Formylanthranilate	0.6360	↑
C00637	Indole-3-acetaldehyde	0.6252	↑

Sphingolipid metabolism	Name	*P*-value	Variation
C00065	L-Serine	0.0214	↓
C00346	Ethanolamine	0.1430	↓
C02934	3-Dehydrosphinganine	0.2193	↓
C01120	Sphinganine 1-phosphate	0.3272	↓
C00319	Sphingosine	0.9850	↓
C00836	Sphinganine<br>Dihydrosphingosine<br>2-Amino-1,3-dihydroxyoctadecane	0.8781	↑

Phenylalanine metabolism	Name	*P*-value	Variation
C00079	L-Phenylalanine	0.0457	↓
C07086	Phenylacetic acid	0.0189	↓
C00601	Phenylacetaldehyde	0.0890	↓
C00166	Phenylpyruvate	0.2016	↓
C00082	L-Tyrosine	0.7697	↓

Phenylalanine, tyrosine and tryptophan biosynthesis	Name	*P*-value	Variation
C00166	Phenylpyruvate	0.2016	↓
C00079	L-Phenylalanine	0.0457	↓

C00082	L-Tyrosine	0.7697	↓

Figure S1 Visualization of creatine pathway and glycine, serine, and arginine metabolism are associated with a network.

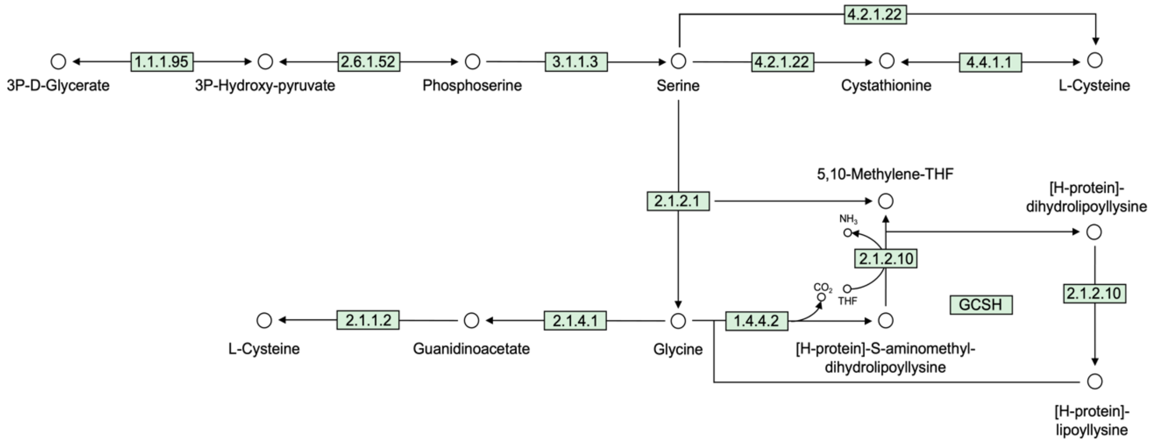

Supplement: Supplementary file 1 [file animals-14-00359-s001.zip › animals-2783731-supplementary.pdf]
